# Modeling of lophotrichous bacteria reveals key factors for swimming reorientation

**DOI:** 10.1038/s41598-022-09823-4

**Published:** 2022-04-20

**Authors:** Jeungeun Park, Yongsam Kim, Wanho Lee, Sookkyung Lim

**Affiliations:** 1 Department of Mathematical Sciences, University of Cincinnati, Cincinnati, OH 45221 USA; 2 Department of Mathematics, Chung-Ang University, Seoul, 06974 Republic of Korea; 3 National Institute for Mathematical Sciences, Daejeon, 34047 Republic of Korea

**Keywords:** Microbiology, Mathematics and computing

## Abstract

Lophotrichous bacteria swim through fluid by rotating their flagellar bundle extended collectively from one pole of the cell body. Cells experience modes of motility such as push, pull, and wrapping, accompanied by pauses of motor rotation in between. We present a mathematical model of a lophotrichous bacterium and investigate the hydrodynamic interaction of cells to understand their swimming mechanism. We classify the swimming modes which vary depending on the bending modulus of the hook and the magnitude of applied torques on the motor. Given the hook’s bending modulus, we find that there exist corresponding critical thresholds of the magnitude of applied torques that separate wrapping from pull in CW motor rotation, and overwhirling from push in CCW motor rotation, respectively. We also investigate reoriented directions of cells in three-dimensional perspectives as the cell experiences different series of swimming modes. Our simulations show that the transition from a wrapping mode to a push mode and pauses in between are key factors to determine a new path and that the reoriented direction depends upon the start time and duration of the pauses. It is also shown that the wrapping mode may help a cell to escape from the region where the cell is trapped near a wall.

## Introduction

Bacterial motility mediated by flagellar rotation is one of the most ubiquitous swimming strategies in the world of microorganisms. Many species of flagellated bacteria navigate the fluid environment as they interact with the physical and chemical microenvironment for biological processes, and their swimming patterns are mostly characterized by the number and the arrangement of flagella over the cell body^1–6^. Among the most studied of bacteria, peritrichous bacteria such as *Escherichia coli* undergo run and tumble modes alternatively^7–9^. When all flagellar motors rotate counterclockwise (CCW), multiple flagella embedded into the cell membrane at random locations come together and form a bundle, and the rotation of the flagellar bundle makes the cell run forward. The cell tumbles and reorients itself upon reversals of at least one or more motors. Monotrichous bacteria such as *Vibrio alginolyticus* undergo run-reverse-flick cycles^5,10–12^. *Vibrio A.* has a single polar flagellum which remains left-handed at all times. When the motor turns clockwise (CW), the flagellum pulls the cell body backward. As the motor reverses from CW to CCW, the cell moves forward briefly and then flicks due to the buckling instability of the relaxed hook. The CCW rotation of the flagellum then pushes the cell body forward in a new direction as the hook becomes loaded again.

Recently, Kühn et al.^3,13^ and Hintsche et al.^2^ independently reported a new swimming pattern in polarly-flagellated bacteria which exhibits three main modes of motility: pull, push, and wrapping modes. Pauses, temporary stops of motor rotation, also occur between the modes. Pull and push modes correspond to the motions when the cell moves backward and forward respectively, which are similar to those of *Vibrio A*. The flagella pull or push the cell body depending on the direction of the motor rotation and the handedness of helical flagella. During a wrapping mode, however, a polar flagellum or a polar flagellar bundle coils around the cell body, and the cell swims in a new direction at a very slow speed, which is distinguished from the conventional run modes. The improved imaging techniques enable to observe the wrapped configuration in some species such as *Pseudomonas putida*^2,14^, *Shewanella putrefaciens*^3,13^, *Helicobacter suis*^15^, *Campylobacter jejuni*^16^, *Burkholderia sp.* and *Aliivibrio fischeri*^17^. It is suggested that there may be more species that adopt the wrapping mode of motility in the course of swimming^4,18–20^. Flagellar wrapping motion has been observed recently and its cause and effect remain an open question. Kühn et al.^3^ first suggested that the wrapping mode may be triggered by an instability of the flagellum under reversal of the motor rotation and a change in the applied torque, and may be useful for escape from complex and structured environments.

In this work, we present a mathematical model of a lophotrichous bacterium which is based on *Pseudomonas putida*^2,14^. A bacterium *P. putida* comprises a rod-shaped cell body and multiple flagella attached near one pole of the cell body. Since all flagella are assumed to work as a flagellar bundle with all motors revolving synchronously, our model contains a single polar flagellum which takes the form of an intrinsically left-handed helix^2,14,21^. We investigate necessary physical conditions to reproduce experimental observations of lophotrichous bacteria that experience combinations of push, pull, and wrapping modes accompanied by pauses in between, and determine key factors for reorientations. We also investigate the hydrodynamic effect of a wall on the cell motility during wrapping and explore the role of the wrapping mode when the bacterium is placed near a solid wall.

## Mathematical methods

**Figure 1:**
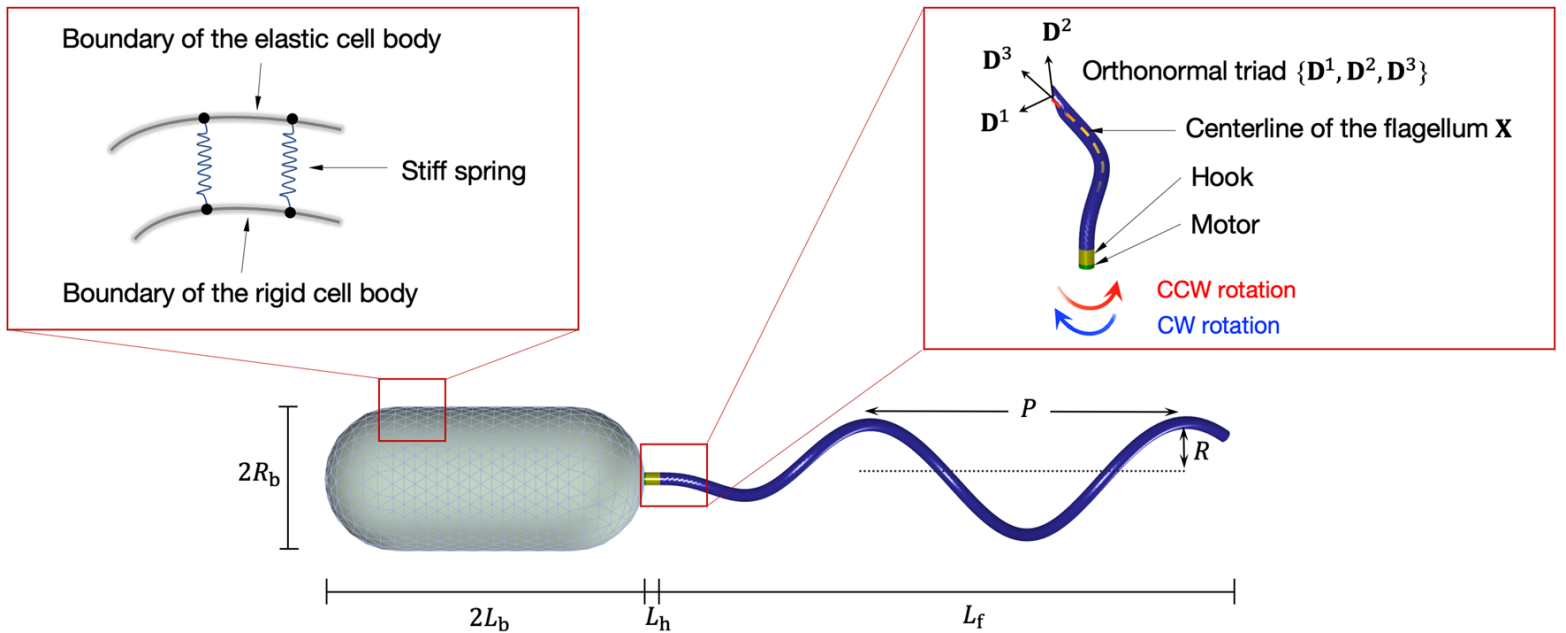
Schematic description of a computational model.

Our mathematical cell model is composed of a rigid cell body and an elastic polar flagellum. The flagellum is assembled by a left-handed helical filament, a compliant hook, and a rotary motor that is embedded into one pole of the cell body, see Fig. 1. In this work, the cell body is enforced to be rigid by a penalty method and the motion of the flagellum can be described by an unconstrained Kirchhoff rod theory. The cell motility is coupled to a surrounding fluid using regularized Stokeslet formulation under the force- and torque-free conditions. The rotary motor generates torque and consequently induces hydrodynamic propulsive forces through flagellar rotation. Physical and computational parameters used in this work are listed in Supplementary Table S1.

First, we describe the dynamics of the cell body which is modelled as a hollow rod-shaped shell and moves as an approximate rigid body. To enforce the rigidity of the cell body, we use two Lagrangian descriptions of the discretized cell body surface; $\mathbf{X}^\mathrm{b}_{i}(t)$ and $\mathbf{Y}^\mathrm{b}_{i}(t)$, $i=1,\dots , n_\mathrm{b}$. The former interacts with the surrounding fluid, while the latter moves as a rigid body. For each *i*, the markers $\mathbf{X}^\mathrm{b}_{i}(t)$ and $\mathbf{Y}^\mathrm{b}_{i}(t)$ are linked by a stiff spring which generates the following force:

1 $$\begin{aligned} {\mathbf{F}}^\mathrm{b}_i(t)=K(\mathbf{X}_i^\mathrm{b}(t)-\mathbf{Y}_{i}^\mathrm{b}(t)), \end{aligned}$$

where *K* is a penalty parameter that determines how tightly they are tied together. This penalty force acts on $\mathbf{Y}^\mathrm{b}_{i}$, and $-{\mathbf{F}}^\mathrm{b}_i(t)$ becomes a body force on the fluid.

The reference configuration of the rigid body is denoted by the time-independent vectors ${\mathbf{Z}}_{i}$ satisfying the condition $\sum _{i=1}^{n_\mathrm{b}} {\mathbf{Z}}_{i}=0$. Then the configuration of the rigid body at time *t*, $\mathbf{Y}^\mathrm{b}_{i}(t)$, is given by

2 $$\begin{aligned} {\mathbf{Y}}_{i}^\mathrm{b} (t) = \mathscr {T}(t) + \mathscr {R}(t) {\mathbf{Z}}_{i}, \quad i=1,\ldots ,n_\mathrm{b}, \end{aligned}$$

where $\mathscr {T}(t)$ is the centroid of {$\mathbf{Y}^\mathrm{b}_{i}$, $i=1,\dots , n_\mathrm{b}$}, and $\mathscr {R}(t)$ is a rotation matrix. Given that $\mathbf{X}^\mathrm{b}_{i}(t)$ is known and that $\mathbf{f}^\mathrm{b}(t)$ and $\mathbf{n}^\mathrm{b}(t)$ are external forces and torques, respectively, acting on the body other than those generated from the coupling springs, the balance equations for the cell body are as follows:

3 $$\begin{aligned} 0 = \mathbf{f}^\mathrm{b} + \sum ^{n_\mathrm{b}}_{i=1}{\mathbf{F}}^\mathrm{b}_i(t),\quad 0 =\mathbf{n}^\mathrm{b} + \sum ^{n_\mathrm{b}}_{i=1}(\mathscr {R}(t) {\mathbf{Z}}_i)\times {\mathbf{F}}^\mathrm{b}_i(t). \end{aligned}$$

Here, the cell body $\mathbf{Y}^\mathrm{b}_{i}(t)$ is assumed to be massless. At each time *t*, we solve Eqs.(1)–(3) for $\mathscr {T}(t)$ and $\mathscr {R}(t)$ to determine $\mathbf{Y}^\mathrm{b}_{i}(t)$. For a detailed description of the solution method, see Lee et al.^22^.

Second, we use Kirchhoff rod theory to describe the dynamics of the helical flagellum which is composed of a rotary motor, a hook, and a filament. The hook is short but more flexible than the filament and bridges the motor and the filament. The Kirchhoff rod can be described in Lagrangian form as $\{\mathbf{X}(s,t), \mathbf{D}^1(s,t),\mathbf{D}^2(s,t),\mathbf{D}^3(s,t)\}$, where $\mathbf{X}(s,t)$ is a three-dimensional space curve and $\{\mathbf{D}^1(s,t),\mathbf{D}^2(s,t),\mathbf{D}^3(s,t)\}$ is its associated orthonormal triad, where *s* is a Lagrangian parameter ranging from $0 \le s \le L$ with $L=L_\mathrm{h} + L_\mathrm{f}$, and *t* is the time. Here, $L_\mathrm{h}$ and $L_\mathrm{f}$ denote the lengths of the hook and the filament, respectively. The reference helical flagellum is defined as ^23^

4 $$\begin{aligned} {\mathbf{X}}_{0}(s) = (r(s) \cos (k s) , r(s) \sin (k s) , s), \end{aligned}$$

where *k* is the wave number. The helical radius *r*(*s*) is zero for $0 \le s \le L_\mathrm{h}$ and $r(s) = R(1- e^{-c (s - L_\mathrm{h})^{2}})$ for $L_\mathrm{h} \le s \le L_\mathrm{h} + L_\mathrm{f}$, where *R* is the radius of the flagellum. We set $c=2$ in order to obtain the helical shape close to that of *P. putida*. We construct the initial configuration of a Kirchhoff flagellum $\mathbf {X}(s,0)$ by attaching ${\mathbf{X}}_{0}(s)$ normally to one pole of the cell body and by setting ${\mathbf{D}}^{3}(s,0)$, ${\mathbf{D}}^{1}(s,0)$, and ${\mathbf{D}}^{2}(s,0)$ to be the unit tangent vector, the principal normal, and binormal vectors to ${\mathbf{X}}(s,0)$, respectively. See Fig. 1.

To describe forces and torques of the filament driven by a rotary motor, we introduce the internal forces and torques that are transmitted across a section of the flagellum, denoted by ${\mathbf{F}}$ and ${\mathbf{N}}$, respectively, and the applied force density and torque density denoted by ${\mathbf{f}}$ and ${\mathbf{n}}$, respectively. Then the balance equations for the linear and angular momenta are given as follows:

5 $$\begin{aligned} 0=\mathbf{f}+\frac{\partial \mathbf{F}}{\partial s} ,\quad 0= \mathbf{n}+\frac{\partial \mathbf{N}}{\partial s}+\frac{\partial \mathbf{X}}{\partial s} \times \mathbf{F}, \end{aligned}$$

where

6 $$\begin{aligned} \mathbf{F}=\sum ^3_{i=1}F_i\mathbf{D}^i,\quad \mathbf{N}=\sum ^3_{i=1}N_i\mathbf{D}^i. \end{aligned}$$

Here, the constitutive relations are as follows:

7 $$\begin{aligned}&F_1 = b_1\mathbf{D}^1\cdot \frac{\partial \mathbf{X}}{\partial s}, \quad F_2 = b_2\mathbf{D}^2\cdot \frac{\partial \mathbf{X}}{\partial s}, \quad F_3 = b_3\left( \mathbf{D}^3\cdot \frac{\partial \mathbf{X}}{\partial s}-1\right) , \end{aligned}$$

8 $$\begin{aligned}&N_1 = a_1\left( \frac{\partial \mathbf{D}^2}{\partial s}\cdot \mathbf{D}^3-\Omega _1\right) , \quad N_2 = a_2\left( \frac{\partial \mathbf{D}^3}{\partial s}\cdot \mathbf{D}^1-\Omega _2\right) , \quad N_3 = a_3\left( \frac{\partial \mathbf{D}^1}{\partial s}\cdot \mathbf{D}^2-\Omega _3\right) , \end{aligned}$$

where {$\Omega _1, \Omega _2, \Omega _3\}$ describes the intrinsic curvature and twist which are determined by the helical radius and pitch of the flagellum. Two bending moduli and twist modulus are given as $a_1$, $a_2$, and $a_3$, respectively, and two shearing coefficients and stretching modulus are given as $b_1$, $b_2$, and $b_3$, respectively. The above constitutive relations can be derived from a variational argument of the energy functional given by

9 $$\begin{aligned} E&=\frac{1}{2}\int \left[ \sum _{i=1}^{3}a_i\left( \frac{\partial \mathbf{D}^j}{\partial s}\cdot \mathbf{D}^k-\Omega _i\right) ^2 + \sum _{i=1}^{3}b_i\left( \mathbf{D}^i\cdot \frac{\partial \mathbf{X}}{\partial s}-\delta _{3i}\right) ^2 \right] ds, \end{aligned}$$

where (*i*, *j*, *k*) is any cyclic permutation of (1,2,3) and $\delta _{3i}$ is the Kronecker delta function. Note that in our simulations, the flagellum is reparametrized by arc length before being used and is discretized with equally spaced points along the curve. Although *s* measures arc length in the reference configuration of the flagellum, it is not arc length, in general, since we use *s* as a Lagrangian (material) coordinate, and our model flagellum is only approximately inextensible. For more detailed description, see Lim et al.^22,24,25^.

Third, we describe how to rotate the motor which is located at one pole of the cell body denoted as ${\mathbf {Y}}^\mathrm{b}_1(t)$. The flagellum $\mathbf {X}$ can be discretized as ${\mathbf {X}}(j\Delta s,t)$, $j=0,\dots ,n_\mathrm{f}$, where $\Delta s$ is the meshwidth that is used in discretizing the Lagrangian parameter *s* of the flagellum and $n_\mathrm{f}$ is the number of marker points along the flagellum. Let $\mathbf {E}(t)$ be the normal direction to the cell surface at ${\mathbf {Y}}^\mathrm{b}_1(t)$. In order to generate a torque by the motor, we apply the following constant torque in the normal direction $\mathbf {E}(t)$ at the ghost point ${\mathbf {X}}(-\Delta s/2,t)$:

10 $$\begin{aligned} \mathbf {N}(-\Delta s/2,t) = -\tau \mathbf {E}(t), \end{aligned}$$

where the motor and hence the flagellum rotate counterclockwise when $\tau > 0$, and clockwise when $\tau < 0$. Note that the torque $\mathbf {N}(-\Delta s/2,t)$ is also applied to the cell body, which results in counterrotation of the cell body.

Additionally, two constraints are imposed at the motor point. One is to keep $\mathbf {Y}^\mathrm{b}_1(t)=\mathbf {X}(0,t)$. The other constraint is to align the tangential direction $\mathbf {D}^3(0,t)$ at $\mathbf {X}(0,t)$ to the normal direction $\mathbf {E}(t)$ of the cell body at ${\mathbf {Y}}^\mathrm{b}_1(t)$. To do that, we apply the feedback moment as follows:

11 $$\begin{aligned} \tilde{\mathbf{n}}(t) =-K_\mathrm{m}(\mathbf {E}(t)\times \mathbf {D}^3(0,t) ), \end{aligned}$$

where $K_\mathrm{m}$ is a large constant. Whenever $\mathbf {E}(t)$ and $\mathbf {D}^3(0,t)$ differ, the restoring moment $\tilde{\mathbf{n}}(t)$ appears to keep them aligned closely. This moment $\tilde{\mathbf{n}}(t)$ is added to the moment $\mathbf{n}(0,t)ds$ at the motor point $\mathbf {X}(0,t)$ in Eq. (13), and the negative moment $-\tilde{\mathbf{n}}(t)$ is also added to the total external torque ${\mathbf {n}}^\mathrm{b}(t)$ acting on the cell body in Eq. (3). For a more detailed numerical method, the reader is referred to the articles^22,24,25^.

Lastly, we couple the cell to the surrounding fluid governed by the viscous incompressible Stokes equations:

12 $$\begin{aligned} 0 = -\nabla p + \mu \Delta \mathbf {u} + \mathbf{g}, \qquad 0 = \nabla \cdot \mathbf {u}, \end{aligned}$$

where the fluid velocity $\mathbf{u}$ and the fluid pressure *p* are unknown functions in terms of the Cartesian coordinates $\mathbf{x}$ and time *t*, and $\mu$ is the fluid viscosity. The regularized force density $\mathbf {g}$ applied to the fluid by the immersed cell is given by

13 $$\begin{aligned} \begin{aligned} \mathbf {g}(\mathbf{x}, t)&= \int _{0}^{L} \big [- \mathbf{f}(s,t) \big ] \psi _{\varepsilon }\big (\mathbf{x} - \mathbf{X}(s,t) \big ) ds +\frac{1}{2} \nabla \times \int _{0}^{L} \big [ - \mathbf{n}(s,t) \big ] \psi _{\varepsilon } \big ( \mathbf{x} - \mathbf{X}(s,t) \big ) ds \\&\quad +\sum _{i=1}^{n_\mathrm{b}} \int _{L^\prime }^{L} \big [- \mathbf{f}^\mathrm{r}_{i} (s,t) \big ] \psi _{\varepsilon }\big (\mathbf{x} - \mathbf{X}(s,t) \big ) ds +\sum _{i=1}^{n_\mathrm{b}}\big [-{\mathbf{F}}^\mathrm{b}_i(t) + \int _{L^\prime }^{L} \mathbf{f}^\mathrm{r}_{i} (s,t) ds \big] \psi _{\varepsilon }(\mathbf{x} - \mathbf{X}_i^\mathrm{b}(t)), \end{aligned} \end{aligned}$$

where the function $\mathbf{f}^\mathrm{r}_{i}(s,t)$ represents the repulsive force to prevent the contact between the flagellum and the cell body and is given by

14 $$\begin{aligned} \mathbf{f}^\mathrm{r}_{i} (s,t) = C \left[ \max \left( \left( 1- \frac{\Vert \mathbf{X}(s,t) - \mathbf{X}_i^\mathrm{b}(t) \Vert }{D} \right) ,0\right) \frac{ \mathbf{X}(s,t) - \mathbf{X}_i^\mathrm{b}(t) }{ \Vert \mathbf{X}(s,t) - \mathbf{X}_i^\mathrm{b}(t) \Vert }\right] , \end{aligned}$$

where *D* is the minimum distance allowed between the flagellum and the cell body and *C* is a sufficiently large constant so that they do not meet. Note that the repulsive force does not activate in the part of the filament close to the motor by choosing the lower limit $L^\prime$ of the integrals greater than 0. We set $L^\prime =15\Delta s=0.4545 \upmu$m throughout this paper. The smooth cut-off function $\psi _{\varepsilon }$ satisfies $\int _{\mathbb {R}^{3}} \psi _{\varepsilon }(\mathbf{r}) d \mathbf{r} = 1$ and is defined by

$$\begin{aligned} \psi _{\varepsilon }(\mathbf{r}) = \frac{15 \varepsilon ^{4}}{ 8 \pi \big ( \Vert \mathbf{r} \Vert ^{2} + \varepsilon ^{2} \big )^{7/2}}, \end{aligned}$$

where $\varepsilon$ is a regularization parameter.

The motion of the flagellum {$\mathbf{X},\mathbf {D}^1,\mathbf {D}^2,\mathbf {D}^3\}$ and of the marker point $\mathbf{X}^\mathrm{b}_i$ that is linked by a stiff spring to the rigid cell body can be described by

15 $$\begin{aligned}&\frac{\partial \mathbf{X}}{\partial t}(s,t) = \mathbf{u} \big (\mathbf{X}(s,t),t \big ) - \frac{1}{\alpha _1} \left[ \mathbf{f} (s,t)+\sum _{i=1}^{n_\mathrm{b}}\mathbf{f}^\mathrm{r}_{i}(s,t) \right] , \quad \frac{\partial \mathbf{X}^\mathrm{b}_i}{\partial t}(t) = \mathbf{u}(\mathbf{X}^\mathrm{b}_{i} (t),t) - \frac{1}{\alpha _2} \left[ {\mathbf{F}}^\mathrm{b}_i(t) - \int _{L^\prime }^{L} \mathbf{f}^\mathrm{r}_{i} (s,t) ds\right] , \end{aligned}$$

16 $$\begin{aligned}&\frac{\partial \mathbf{D}^{j}}{\partial t}(s,t) = \left[ \mathbf{w} \big (\mathbf{X}(s,t),t \big ) - \frac{1}{\beta } n_{3}(s,t) \mathbf{D}^{3}(s,t) \right] \times \mathbf{D}^{j}(s,t) , \qquad j=1,2,3, \end{aligned}$$

where $\alpha _i$’s and $\beta$ are translational and rotational drag coefficients, respectively, and $n_{3}(s,t)$ is the axial component of torque density $\mathbf{n}$. We allow linear and angular slips between the cell and the fluid so that these slip conditions facilitate the wrapping mode of motility^25^. The angular velocity of the fluid $\mathbf{w}$ is defined as

17 $$\begin{aligned} \mathbf{w} (\mathbf{x}, t) = \frac{1}{2} \nabla \times \mathbf{u} (\mathbf{x} ,t). \end{aligned}$$

## Results

Swimming modes of bacteria are closely related to their physical and geometrical properties. We first explore the dependence of the modes of motility on the hook’s bending modulus and the applied torque generated by the flagellar motor. Then we determine proper ranges of the parameter values to reproduce the experimentally observed swimming motion of lophotrichous bacteria such as *P. putida*. Bacteria reorient in search of nutrients or away from hazardous substances. We investigate the effect of various transitions of swimming modes that lophotrichous bacteria may experience in the course of swimming by measuring the reoriented swimming direction. We also study the role of wrapping motion when the bacteria swim near a wall. Throughout this paper, the flagellum is assumed to be left-handed. Thus, the positive (negative) value of $\tau$ induces CCW (CW) rotation of the flagellum, in which case the rotational frequency *f* of the motor is defined to be positive (negative). We also define the forward swimming speed $V_f$ as the velocity of the cell body in the direction from the motor point to the opposite pole of the cell body, i.e., $V_f(t)=-\frac{d\mathscr {T}}{dt}\cdot \mathbf {E}(t)$.

### Classification of swimming modes

**Figure 2:**
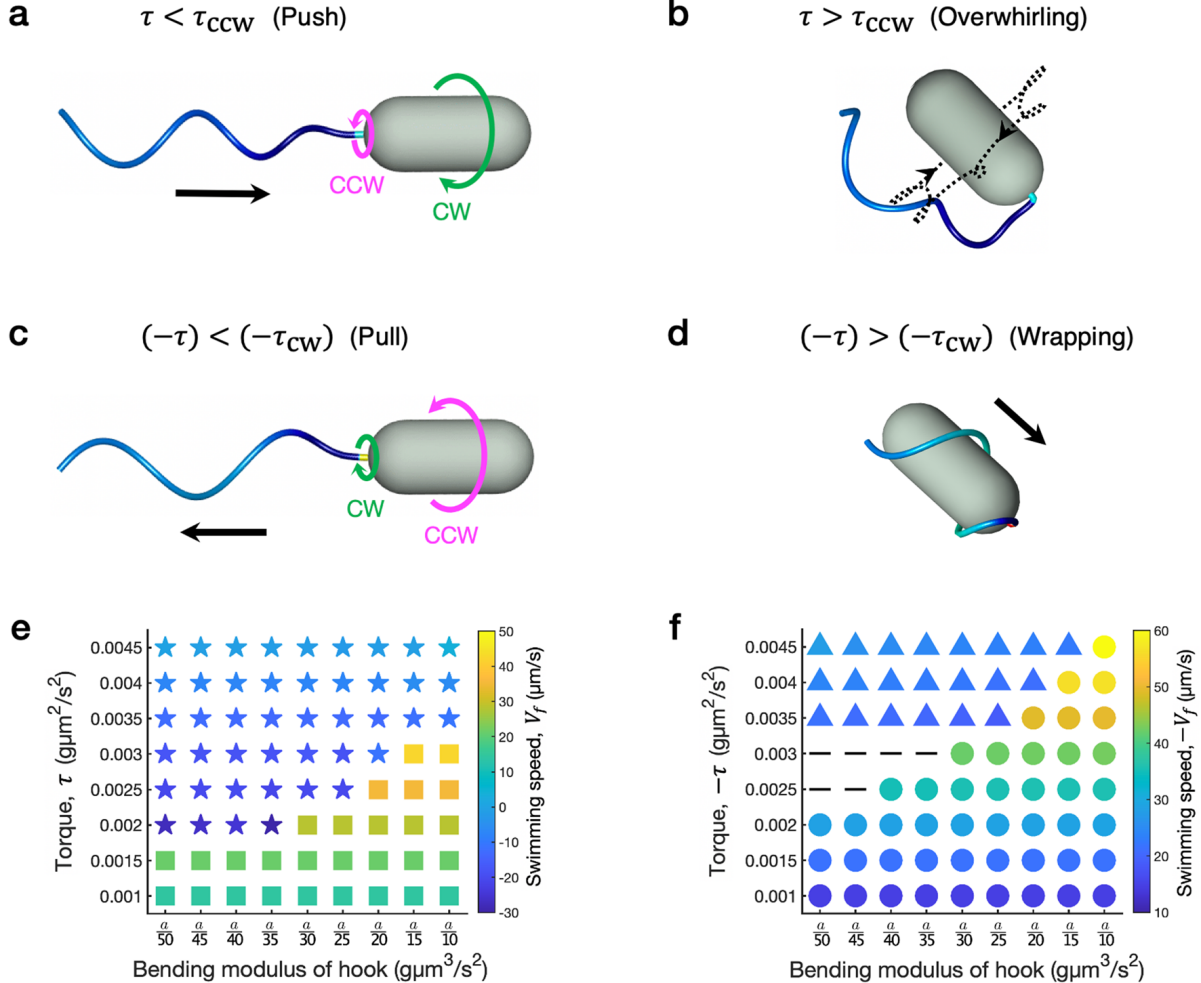
Stable swimming modes of a polarly-flagellated bacterium. The helical filament is intrinsically left-handed and the cell displays four stable swimming modes as the motor rotates either CCW (**a**,**b**) or CW (**c**,**d**), and these stable swimming modes can be further classified as functions of the bending modulus of the hook ($a^\mathrm{hook}$) and the magnitude of applied torque ($|\tau |$) when the motor rotates either CCW (**e**) or CW (**f**). Different shapes of markers represent the push ($\blacksquare$), overwhirling ($\bigstar$), pull ($\bullet$), and wrapping ($\blacktriangle$) modes, and the imaginary separating lines of these marker shapes determine the critical thresholds of the applied torque, $\tau _\mathrm{cw}$ and $\tau _\mathrm{ccw}$. The markers ($\varvec{-}$) indicate that the bacterium shows neither wrapping nor pull mode for a given simulation time because the hook is too flexible and the applied torque is close to the critical value. Colors indicate the average swimming speeds measured for each mode of motility after the simulation reaches the stable steady motion.

We classify swimming patterns of a polarly-flagellated bacterium as we vary the bending rigidity of the hook ($a^\mathrm{hook}$) and the magnitude of applied torque ($|\tau |$), while the motor and hence the flagellum turn either CCW ($\tau > 0$) or CW ($\tau < 0$). The rest of the parameter values are adopted from Supplementary Table S1. As the motor generates the torque, the motor twist is transmitted to the filament through the hook, which rotates the whole flagellum and propels the cell body so that the bacterium reaches a steady motion after some time. Figure 2 shows four different configurations in stable swimming modes (a–d) and the classification of the swimming modes as functions of the bending modulus of the hook and the magnitude of applied torque when the motor rotates either CCW (e) or CW (f).

When the motor rotates CCW ($\tau > 0$) and the bending modulus of the hook is set to a certain value ranging from *a*/50 to *a*/10, where $a=0.003$ g$\upmu$m$^{3}/$s$^{2}$ is the constant bending modulus of the flagellar filament, there exists a critical size of torque $\tau _\mathrm{ccw}$ that separates the *overwhirling* mode from the *push* mode, see Fig. 2a,b,e and Supplementary Video S1. For small torque $\tau < \tau _\mathrm{ccw}$, the bacterium is in the push mode in which the cell swims forward with $V_f>0$. For large torque $\tau > \tau _\mathrm{ccw}$, however, the bacterium goes through the overwhirling motion in which the flagellum experiences a large excursion and eventually bends toward the cell body due to the flagellar instability and rotates around its rotation axis in a steady state. Note that, in the case of overwhirling, the forward swimming speed $V_f$ is negative near the threshold $\tau _\mathrm{ccw}$ and then increases up to be positive as the applied torque $\tau$ increases, see Supplementary Fig. S1.

When the motor rotates CW ($\tau < 0$), there is also a critical torque size of $\tau _\mathrm{cw}$ that separates the *wrapping* mode from the *pull* mode for each given value of the bending modulus of the hook, see Fig. 2c,d,f and Supplementary Video S1. The pull mode in which the flagellum pulls the cell body so that the bacterium moves backward ($V_f<0$) occurs when the applied torque size $(-\tau )$ is less than $(-\tau _\mathrm{cw})$. When $(-\tau ) > (-\tau _\mathrm{cw})$, the bacterium shows the wrapping motion in which the polar flagellum buckles and coils around the cell body in the shape of a corkscrew and rotates in a steady manner. Note that when the hook is very flexible and the applied torque is near the threshold, it might take a while for the bacterium to reach a stable motion. The dash markers in Fig. 2f are such cases in which the motion is not identified for the given simulation time in this work.

**Figure 3:**
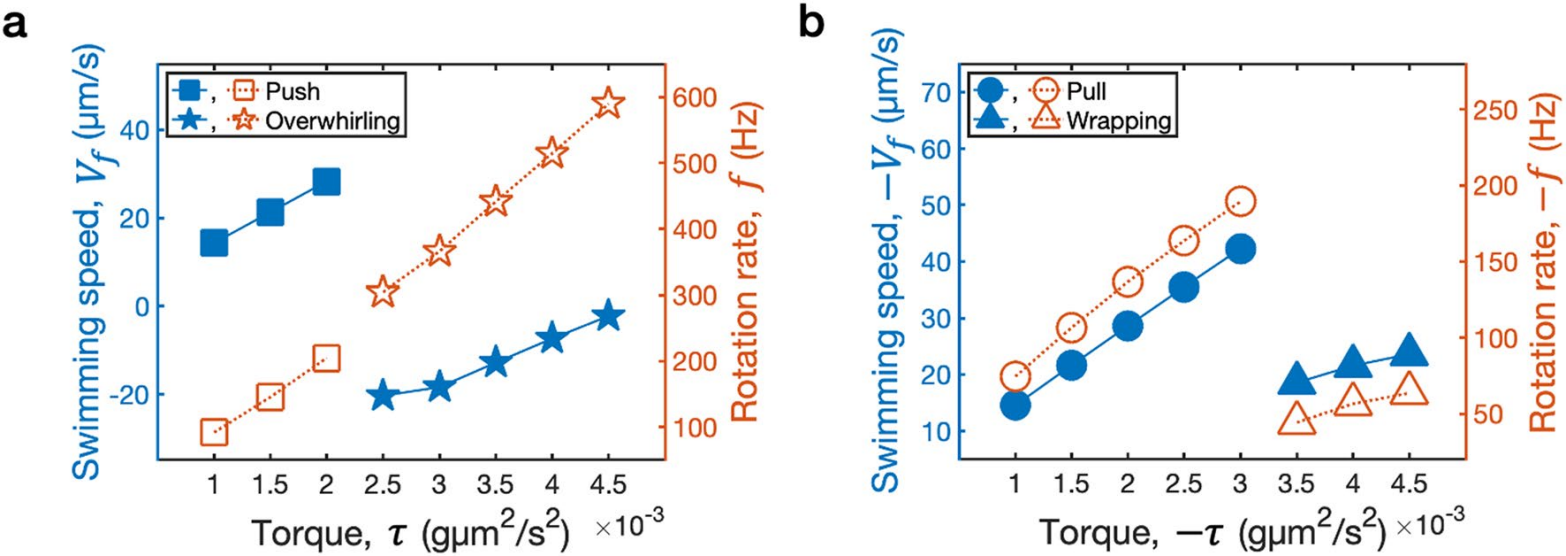
Average swimming speeds and the motor rotation rates as functions of the magnitude of applied torque when the motor rotates either CCW (**a**) or CW (**b**). The bending moduli of the filament and the hook are fixed at $a=0.003$ g$\upmu$m$^{3}/$s$^{2}$ and $a^\mathrm{hook}=a/25=0.00012$ g$\upmu$m$^{3}/$s$^{2}$, respectively.

It is shown in Fig. 2e,f that, regardless of the direction of motor rotation, both thresholds $\tau _\mathrm{ccw}$ and $-\tau _\mathrm{cw}$ have a similar tendency to increase monotonically as $a^\mathrm{hook}$ increases. Higher torque sizes than the thresholds cause a buckling instability and thus result in large deviations of the flagellum whether the motor rotates CCW or CW. In both push and pull modes, the swimming speed increases as the applied torque size gets larger while the bending modulus of the hook is being held fixed. When the applied torque is fixed, on the other hand, the swimming speed remains almost the same as the bending modulus of the hook changes. Figure 3 illustrates the motor rotation rates and the average swimming speeds as functions of the applied torque when the motor rotates either CCW (a) or CW (b). The bending moduli of the filament and the hook are set as $a=0.003$ g$\upmu$m$^{3}/$s$^{2}$ and $a^\mathrm{hook}=\frac{a}{25}=0.00012$ g$\upmu$m$^{3}/$s$^{2}$, respectively. The average swimming speed and the motor rotation rate are approximately proportional to the magnitude of applied torque with discontinuities at the thresholds $\tau _\mathrm{ccw}$ (a) and $\tau _\mathrm{cw}$ (b). Across the thresholds, the swimming speed and the motor rotation rate dramatically drop except for the rotation rate in CCW motor rotation. These linear relationships with jumps at the thresholds are true for other values of the bending modulus of the hook, see Supplementary Fig. S2.

For different values of the bending modulus *a* of the flagellar filament, swimming patterns appear to be similar to those in Fig. 2 except that the two thresholds $\tau _\mathrm{ccw}$ and $-\tau _\mathrm{cw}$ are shifted upward as the bending modulus of the filament increases, see Supplementary Fig. S3. It is worth mentioning that, if the bending moduli of both the filament and the hook are sufficiently large, the model organism only shows either pull or push mode depending on the direction of motor rotation. If the flagellum is too flexible, the motor twist is not properly transmitted to the filament, and hence the swimming modes reported above may not be sustained. We also investigate the effect of the hook length on the swimming modes, while the rest of the parameters are fixed as in Supplementary Table S1. Our simulations show that the flagellum with a longer hook is more likely to be deformed, which facilitates the overwhirling or wrapping mode as the motor turns CCW or CW, respectively, see Supplementary Table S2. However, the flagellum with a shorter hook tends to remain in the push or pull mode.

The classification of swimming modes suggests that the model organism needs appropriate ranges of physical and geometrical properties of the flagellum in order to achieve experimentally observed swimming motions. We have chosen the default values for the computational model of lophotrichous bacteria such as *P. putida* including the applied torque and the bending rigidities of the filament and the hook for each swimming mode so that the resultant swimming speeds and the order of magnitude of motor rotation rates for each mode are in good agreement with experimental data^2,14^. Note in particular that the bending modulus of the filament is in the similar range of that of *E. coli*^26^.

### Modes of motility of lophotrichous bacteria: pull, wrapping, push with a pause

**Figure 4:**
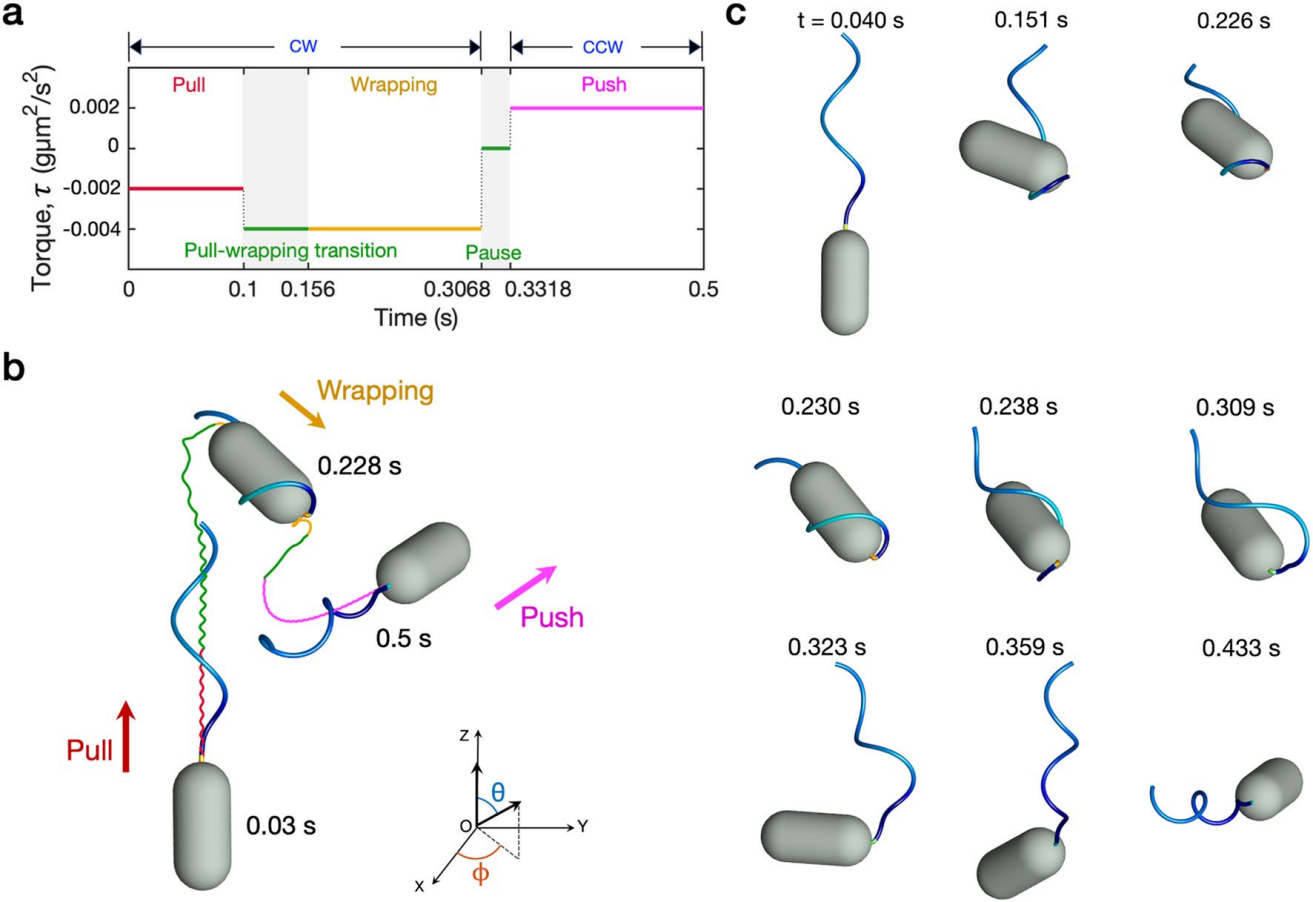
A series of swimming modes in the order of pull, wrapping, pause, and push modes. Time evolution of the applied torque $\tau$ corresponding to each mode (**a**), trajectory of the centroid of the cell body together with three snapshots of the cell in the pull, wrapping, and push modes (**b**), and snapshots of the cell at different times (**c**). Different colors of the trajectory in (**b**) represent the different modes of swimming; pull (red), wrapping (orange), transition/pause (green), and push (magenta).

Lophotrichous bacteria such as *P. putida* typically display combinations of three swimming modes of motility; push, pull, and wrapping modes. They often exhibit pauses in between by stopping the motor rotation^2,14,21,27^. We here consider a cell that experiences a series of swimming modes in the order of pull, wrapping, pause, and push modes, see Fig. 4, Supplementary Fig. S4 and Video S2. Figure 4a shows time evolution of the applied torque $\tau$ corresponding to each mode. Note that the transition time between pull and wrapping modes represents the time taken to evolve into a complete wrapping mode, which is consistent with experimental data^2,14^. Figure 4b displays a trajectory of the centroid of the cell body together with three configurations of the cell in pull, wrapping, and push modes. These changes of swimming modes allow the cell to reorient. Figure 4c illustrates a time evolution of snapshots of the cell motility when the applied torque is given in Fig. 4a.

Among the modes of motility, the wrapping mode is shown to play a significant role in reorientation of swimming direction of polarly-flagellated bacteria, since the flagellum without chiral transformation can pull or push the cell body only in a straight line depending on the direction of the motor rotation. Our simulations also demonstrate that pauses allow the cell to make smooth transitions from one mode to another, in particular, when the motor switches the direction of rotation from CW to CCW. Without a pause, it may fail to switch the mode from wrapping to push. We have observed that a sudden reversal of the motor without a pause interrupts the unwrapping process of the flagellum from the cell body and leads the cell to go through the overwhirling motion instead of the push motion. During the pause, the flagellum becomes relaxed and is aligned to the axis of the cell body so that the cell is ready to be in the push mode.

### Bacterial reorientation

Experiments using cell tracking devices have shown that lophotrichous bacteria can alter the swimming course by switching the swimming mode from one to another or by stopping the motor rotation temporarily^2,14,20,21,27,28^. The previous studies on the swimming courses, however, have relied mostly on 2D cell tracking results. In order to provide more concrete insight into reorientations, we investigate the reoriented direction when the cell switches from one mode to another by measuring the turn angle (or latitude) and longitude of the swimming direction of the switched mode from that of the original mode. See the inlet of Fig. 4b in which the positive *z*-direction is the swimming direction of pull as the original mode; however, the original mode can be changed. Note that the choice of *x*, *y* axes within the plane orthogonal to the *z*-axis is arbitrary.

**Figure 5:**
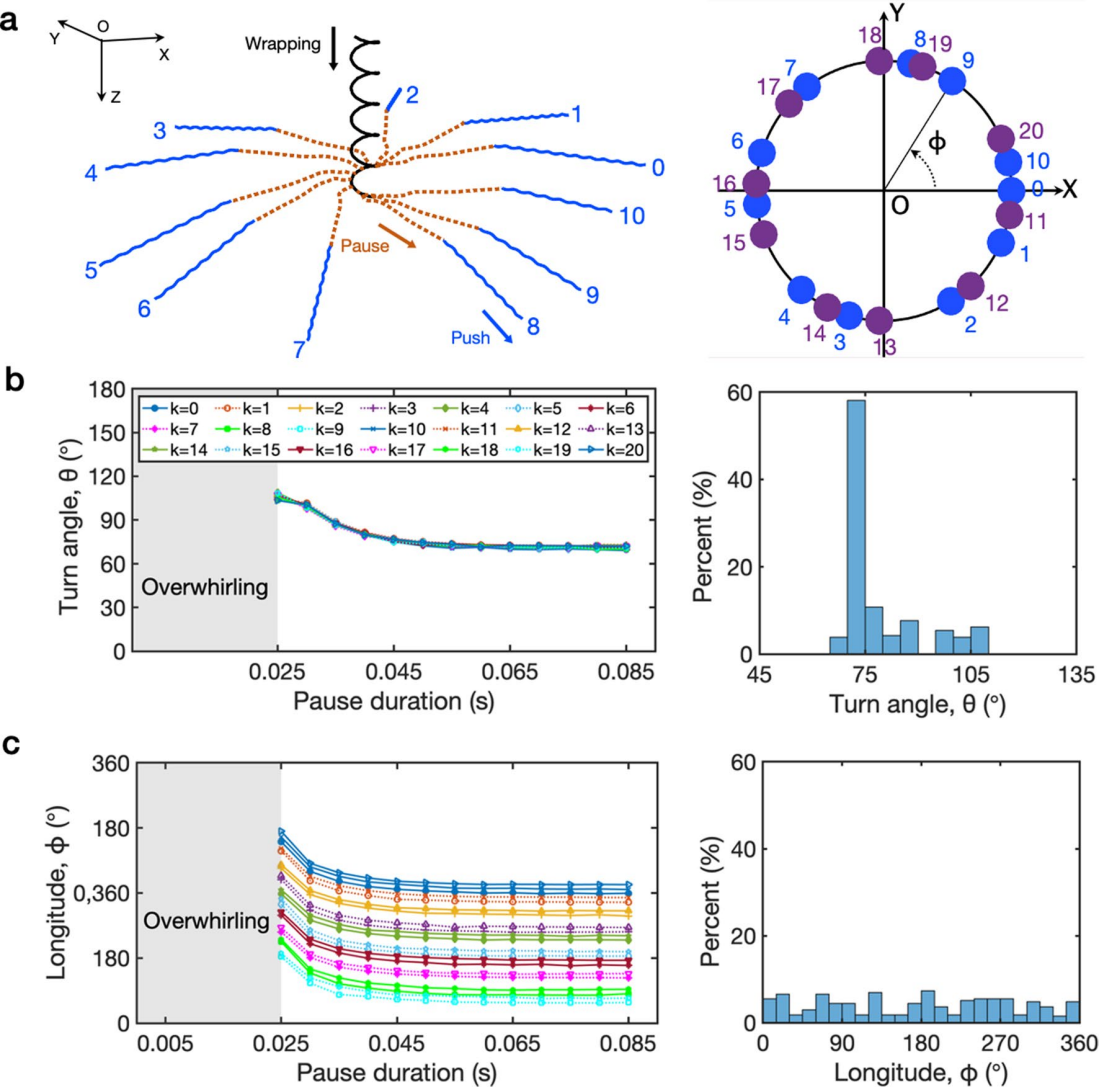
Cells’ reorientation during ‘wrapping-pause-push’ transformations. Reorientations in terms of turn angle and longitude are determined by combinations of pause initiation time $\mathscr {P}_\mathrm{I}^k=(0.15+0.0017k)$ s, $k=0, \ldots ,20$, and pause duration $\mathscr {P}_\mathrm{D}^j=(0.025+0.005j)$ s, $j=0, \,.\,.\,, 12$, where 0.017 s corresponds to the period of one cycle of flagellar rotation measured during the wrapping mode. Therefore, the potential pause initiation time is uniformly distributed over the time interval corresponding to two cycles of flagellar rotation. (**a**) Displays 11 individual trajectories (left) of the cell center, where a pause is initiated with $\mathscr {P}_\mathrm{I}^k$, $k=0, \ldots ,10$, and the longitudes $\phi$ with $\mathscr {P}_\mathrm{I}^k$, $k=0, \ldots ,20$ (right), while the pause duration is fixed as $\mathscr {P}^7_\mathrm{D}=0.06$ s. Cells move downward drawing helical curves (solid black) and tumble at different pause initiation times (dotted red) and then push forward (solid blue). Notice that the longitude makes approximately two turns in clockwise direction against pause timing corresponding to two cycles of flagellar rotation, see the right panel in (**a**) where each turn is distinguished by colors. The turn angles (**b**) and longitudes (**c**) of 273 events with all the combinations of $\mathscr {P}_\mathrm{I}^k$ and $\mathscr {P}_\mathrm{D}^j$ are drawn together with the collective histograms in the right panels. When the pause duration is less than 0.025 s, the cell displays the overwhirling motion rather than the push mode. For a fixed pause duration, the turn angles do not depend on pause initiation time but the longitude does. When the pause initiation time is fixed, the turn angle and the longitude converge to certain values as the pause duration increases. Whereas the turn angles form a unimodal distribution with a peak at $70^\circ$–$75^\circ$ with the mean ± standard deviation being equal to $78.7^\circ \pm 11.31^\circ$, longitudes spread out ranging from $0^\circ$ to $360^\circ$ and show no trend.

Figure 5 shows cells’ reorientation during ‘wrapping-pause-push’ transformations as pause initiation time and duration are varied. The turn angle and longitude are determined by combinations of pause initiation time $\mathscr {P}_\mathrm{I}^k=(0.15+0.0017k)$ s, $k=0, \ldots ,20$, and pause duration $\mathscr {P}_\mathrm{D}^j=(0.025+0.005j)$ s, $j=0, \,.\,.\,, 12$, where 0.017 s corresponds to an approximate period of one cycle of flagellar rotation measured during the wrapping mode. Therefore, the potential pause initiation time $\mathscr {P}_\mathrm{I}^k$ is uniformly distributed over the time interval corresponding to two cycles of flagellar rotation. Figure 5a displays 11 trajectories of the cell center for $k=0, \ldots ,10$ (left panel) and the longitudes for $k=0, \ldots ,20$ (right panel), while the pause duration is fixed as $\mathscr {P}_\mathrm{D}^7=0.06$ s. Numbers in Fig. 5a indicate the value of *k* which determines the pause start time. Each cell draws a helical path during the wrapping mode (solid black), then tumbles during the pause (dotted red), and then draws roughly a linear path which corresponds to the steady push mode (solid blue), see Supplementary Video S3. The turn angle and the longitude can be measured from the axis of the helical path of the steady wrapping mode to the linear path of the steady push mode. Note that while the turn angle remains constant due to the fixed pause duration, the longitude in the right panel makes one turn in clockwise direction against the distribution of pause timing for each period of flagellar rotation, which is distinguished by colors, and the average difference in longitude between two consecutive pause initiation times is $34.8^\circ \pm 10.9^\circ$ (Mean ± SD).

Figure 5b,c displays turn angles (b) and longitudes (c) of 273 events resulted from all combinations of the pause initiation time $\mathscr {P}_\mathrm{I}^k$, $k=0, \ldots ,20$, and the pause duration $\mathscr {P}_\mathrm{D}^j$, $j=0, \,.\,.\,, 12$. For a fixed pause duration, the turn angles do not depend on pause initiation time but the longitude does. When the pause initiation time is fixed, the turn angle and the longitude converge to certain values as the pause duration increases. Whereas the limiting turn angle is a constant, the limiting values of longitude are different depending on the pause initiation time. Thus, the statistical behaviors shown in histograms in the right panels are summarized as follows; whereas the turn angles form a unimodal distribution with a peak at $70^\circ$–$75^\circ$ with the mean ± standard deviation being equal to $78.7^\circ \pm 11.31^\circ$, longitudes spread out ranging from $0^\circ$ to $360^\circ$ and show no trend in the 273 cases. Note that when the pause duration is less than 0.025 s, the cell displays the overwhirling motion rather than push mode, which suggests that the wrapped cell needs enough pause time to transform to push mode. Note also that, when the pause duration is sufficiently long, the turn angle remains the same. As a result, the turn angle distribution may be influenced by how long bacteria take a pause on average, which may differ across species.

When the cell goes through ‘wrapping-pause-wrapping’ transformation, turn angles form a multimodal distribution ranging from $0^\circ$ to $145^\circ$, and longitudes make approximately uniform distribution spreading out from $0^\circ$ to $360^\circ$, see Supplementary Fig. S5. Similar to those in the above ‘wrapping-pause-push’ transformation, turn angles remain a constant independent of the pause initiation time for each pause duration, and longitudes are characterized by pause initiation time. When the cell takes ‘wrapping-pause-pull’ transformations, the statistics of turn angles show a unimodal distribution ranging from $105^\circ$ to $165^\circ$ peaked at $110^\circ$, while the longitudes are spread out across the whole range, see Supplementary Fig. S6. The overall patterns of the distributions of turn angles and longitudes are very similar to those in the case of ‘wrapping-pause-push’ modes.

Cells considered so far in this section switch from wrapping to another mode. Now we consider reverse situations: ‘push-pause-wrapping’ and ‘pull-pause-wrapping’ modes, see Supplementary Fig. S7. It is very intriguing that the turn angles in both cases show unimodal distributions with peaks being centered around $40^\circ$–$45^\circ$ for the former and $135^\circ$–$140^\circ$ for the latter, which makes up each other for $\sim 180^\circ$. This may be because cells swim in the opposite directions in pull and push modes before switching to wrapping via a pause. The longitudes in both cases show a similar pattern of multimodal distributions. Note that, in the absence of the wrapping motion, any combinations of push, pull, and pause lead the cell to move in a straight line resulting in turn angles of either $0^\circ$ or $180^\circ$. These simulation results are natural and in good agreement with experimental data^2,14^.

### Effect of a solid surface on bacterial behavior in the wrapping mode

Lophotrichous bacteria are often found in confined or complex environments, and experimental observation suggests that the wrapping motion is a beneficial swimming strategy in their natural habitats^2,3,17^. Whereas pull and push motions in the vicinity of solid surfaces have been studied experimentally, theoretically, and computationally^29–32^, the hydrodynamic effect of solid surfaces on the wrapping motion has not been studied well. Here, we investigate the swimming behavior when the cell goes through the wrapping motion near a planar wall, which may help to understand benefits of wrapping motion in a structured environment. In order to describe the hydrodynamic interaction of the cell near the planar wall which is $z=0$, we incorporate the method of image system into our mathematical model, refer to Cortez et al.^33^ and Park et al.^32^ for details on the method. In addition, we prevent contact between the wall and the cell by using the repulsive force $\mathbf{f}^\mathrm{r}_\mathrm{w}(s,t)= c_\mathrm{w}\,\mathrm{max}( 1.0-\tilde{X}_{3}/D_\mathrm{w},\,0)\, \mathbf{e}_3$, where $c_\mathrm{w}=2$ g/s$^2$ is a stiffness constant, $\mathbf{e}_3$ is the positive *z*-directional unit vector, $\tilde{X}_{3}$ is the third component of the flagellum $\mathbf{X}(s,t)$ or the cell body $\mathbf{X^\mathrm{b}}(t)$, and $D_\mathrm{w}=$0.09 $\upmu$m is the minimum distance allowed from the wall to the cell. The repulsive force acts on the cell when the distance between the wall and the cell is less than $D_\mathrm{w}$. Throughout this section, we choose the cell model with the parameters given in Supplementary Table S1 and put it in parallel to the wall with $h_0$ being the initial height of the cell body center from the wall.

**Figure 6:**
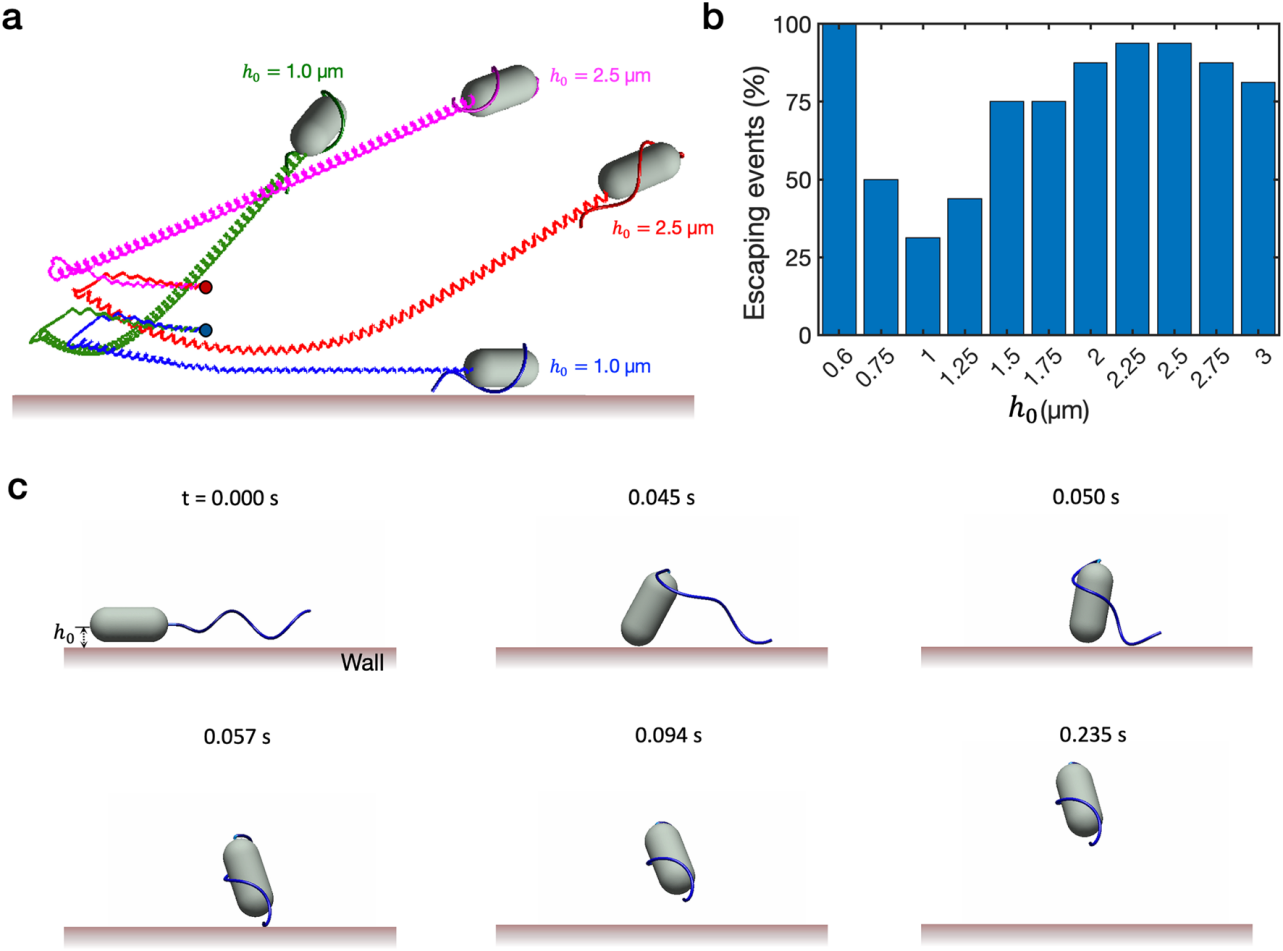
Hydrodynamic effect of the wall on the cell movement in the wrapping mode. (**a**) Trajectories of cells in the wrapping mode for four combinations of two different initial cell rotation angles (180$^\circ$, 337.5$^\circ$) and two different initial heights ($h_0=1.0, ~2.5$ $\upmu$m), (**b**) displays percentage of escaping events as a function of $h_0$ for various initial cell rotation angles, and (**c**) illustrates snapshots of one escaping event when $h_0=0.6$ $\upmu$m.

First we set $\tau =-0.004$ g$\upmu$m$^{2}/$s$^{2}$ for all times, which induces the cell to go through the transition period to the wrapping mode accompanied by a reorientation and then to swim steadily in the resultant direction. This resultant direction is affected by the initial rotation of the cell about the major axis of cell body and the initial height, which may determine the upward or downward departure of the wrapped cell at an early stage. Figure 6a illustrates four trajectories of cells in the wrapping motion, resulted from the combinations of two different initial rotation angles and two different initial heights, see Supplementary Video S4. In the early stage of the cell movement, upon completion of the wrapping after the transition period, the pole of the wrapped cell, at which the motor is attached, points either upward or downward. Our simulations show that if the wrapped cell points upward at an early stage, it continues to swim away from the wall (‘escaping’); however, if the wrapped cell points downward at an early stage, the cell can either escape from the wall or be trapped near the wall depending on $h_0$. Figure 6b displays percentage of escaping events as a function of $h_0$ for various initial cell rotation angles. When 0.75 $\upmu$m $\le h_0 \le$ 1.25 $\upmu$m, both escaping and trapped events occur almost equally. If $h_0$ is large enough, most of the cells swim freely away from the wall. It is interesting to see that the cell always escapes when $h_0 \le$ 0.6 $\upmu$m, in which case the wall pushes away the motor point at which the instability of flagellum occurs during transition and the wrapped cell points and swims upward, see Fig. 6c for snapshots at different times.

**Figure 7:**
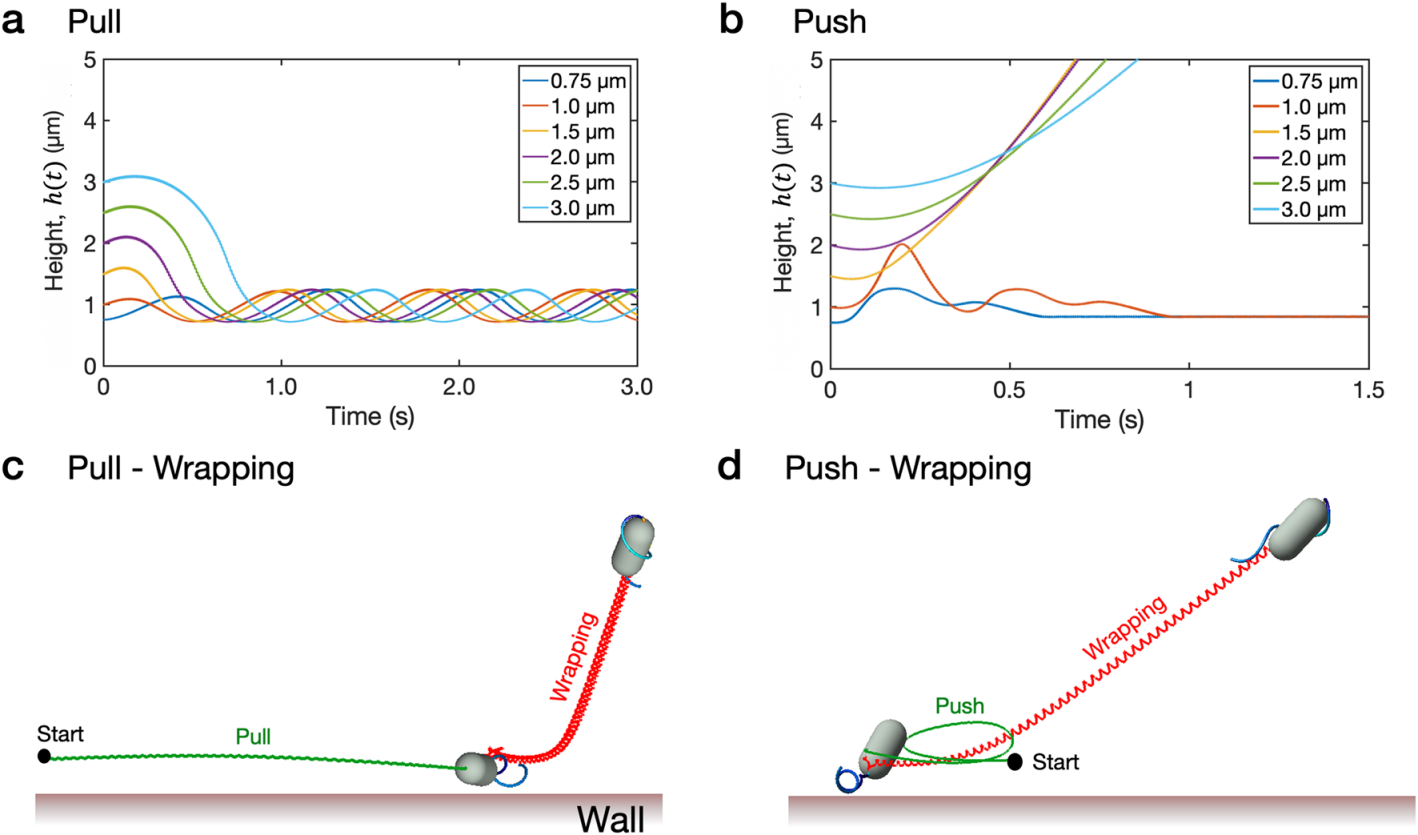
Escaping events via wrapping. Time evolutions of the height *h*(*t*) of cell body in pull (**a**) and push (**b**) modes with different initial heights, and two escaping cases via a wrapping mode; ‘pull-wrapping’ (**c**) and ‘push-wrapping’ (**d**). The initial location of the cell in (**c**,**d**) is marked as a filled circle ($\bullet$) and $h_0=1$ $\upmu$m.

Our simulation results above demonstrate that a majority of the cells in the wrapping mode tend to escape from the wall. However, the cells in the pull mode with a constant $\tau =-0.002$ g$\upmu$m$^{2}/$s$^{2}$ swim up and down periodically with a constant amplitude in the long run, whereas the cells in the push mode with $\tau =0.002$ g$\upmu$m$^{2}/$s$^{2}$ show both escaping and trapped events separated by the critical initial height $h_0=1.1$ $\upmu$m. Figure 7a,b display time evolutions of the cell height for each mode. As compared to the cell in the pull mode, the cell in the push mode is likely to escape as long as the initial height is large enough. When the cell in the push mode is too close to the wall, its tail can be trapped to the wall preventing the cell from taking off, see the green line in Fig. 7d. The common observation made in both push and pull modes is that when the cell is positioned very close to the wall, it can escape through neither push nor pull. Thus the cell trapped to the wall might utilize the wrapping mode to escape. Figure 7 demonstrates two escaping cases via wrapping: ‘pull-wrapping’ (c) and ‘push-wrapping’ (d), see Supplementary Video S5.

## Summary and discussion

Our model organism in this work is inspired by lophotrichous bacteria^2,3,14,15,17^ that is composed of a rigid rod-shaped cell body and a tuft of multiple flagella extended from one pole of the cell body. Since all motors of flagella in a tuft are assumed to be synchronized^2,34,35^, our model organism has one polar flagellum whose helical filament is intrinsically left-handed. Our simulations show that the flagellar flexibility, the magnitude of motor torque, and the direction of motor rotation play a major role in determining bacterial swimming modes which are classified into push, pull, wrapping, and overwhirling. Our model suggests necessary conditions of physical and geometrical properties of the flagellum in order for the cell to reproduce the experimentally observed data in *P. putida*, which may also be applied and adjusted to different species.

Lophotrichous bacteria such as *P. putida* reorient by switching the swimming mode from one to another with or without temporal pauses of the motor rotation. In the absence of the wrapping mode in the course of swimming, the cell can take only the unidirectional runs; however, wrapping accompanied by pauses allows the cell to take different paths. In particular, temporary pauses of motor rotation play an important role in determining the new swimming direction which depends on the initiation time and duration of pause. When the wrapping mode is followed by a pause and then switched to a different swimming mode, our simulations demonstrate that the turn angle before and after the pause is determined by the pause duration but is independent of the pause initiation time. Unlike the turn angle, the longitude shows a cyclic pattern varying from $0^\circ$ to $360^\circ$ as the pause initiates at different times. In fact, the longitude cycles with the phase of flagellar rotation. The similar phenomenon has been observed in peritrichous bacteria such as *E. coli*, which is achieved by varying initiation time and duration of tumble^22^. Quantifying reorientations of individual cells is important in understanding collective behavior of cells in their natural environment. The pattern of spreading and migration of cells at the population level is often modeled by a diffusion-mediated transport equation where the diffusion is described on the basis of the distributions of turn angles and run times of individual cells. Therefore, our simulation results may give insight into the motility pattern at the population level^2,14^.

It is known experimentally and computationally that swimming paths of cells near physical barriers such as rigid surfaces can be modified. For example, under the same cell conditions, cells running forward or backward in a free space without any physical obstacles turn into circular motions in the presence of walls and stay close to the wall^2,29–32^. However, cells in a steady wrapping mode always draw a linear path regardless of the existence of walls. What is important for reorientation is that the cell body turns as it enters into the wrapping mode. In the wrapping mode, the cell body counterrotates to the flagellum which wraps around the cell body, and the resultant flow field induces a straight swimming trajectory. This suggests that when the cell is in structured confinements, the wrapping mode may be utilized to escape from the trap zone and allows the cell to pass through passages and to navigate towards nutrients located away from the confinement. In fact, some species of bacteria tend to have wrapping modes as they are located in a confined space^3,15,17^. When such bacteria are trapped at an interface that increases the load exerted on the flagella, cells transition into the wrapping state, which may help move through narrow spaces^3^. It is, however, worth mentioning that the mechanism that triggers the wrapping of the cell is not known yet.

In this work, we assume that all flagellar motors behave synchronously so that we can represent a tuft of flagella as a single flagellar bundle. However, it is reported that the direction of rotation of flagellar motors is not always synchronized^2,14^. Our model presented here can be extended to include multiple flagella, and their non-synchronous motor reactions can be demonstrated by employing the chemosensory system and the intracellular signaling pathway^36^, which will be our future work. Since most experimental studies on lophotrichous bacteria are limited to a two-dimensional setting, our three-dimensional computational approach may help to better understand the swimming motility in response to external stimuli and in the context of natural habitats.

## Supplementary Information

Supplementary Information 1.

Supplementary Information 2.

Supplementary Information 3.

Supplementary Information 4.

Supplementary Information 5.

Supplementary Information 6.
