# Supplementary material for: Modeling of lophotrichous bacteria reveals key factors for swimming reorientation
Source: Sci Rep. 2022 Apr 20;12:6482. doi: 10.1038/s41598-022-09823-4 (PMC9021275; doi:10.1038/s41598-022-09823-4)
Supplement: Supplementary file 1 — Supplementary Information 1. [file 41598_2022_9823_MOESM1_ESM.pdf]

Supplementary Information

Modeling of lophotrichous bacteria reveals key factors for swimming reorientation

Jeungeun Park[1], Yongsam Kim[2,*], Wanho Lee[3], and Sookkyung Lim[4,*]

[1,4]Department of Mathematical Sciences, University of Cincinnati, Cincinnati, OH 45221, USA
[2]Department of Mathematics, Chung-Ang University, Seoul, 06974, Republic of Korea
[3]National Institute for Mathematical Sciences, Daejeon 34047, Republic of Korea
[*]sookkyung.lim@uc.edu or kimy@cau.ac.kr

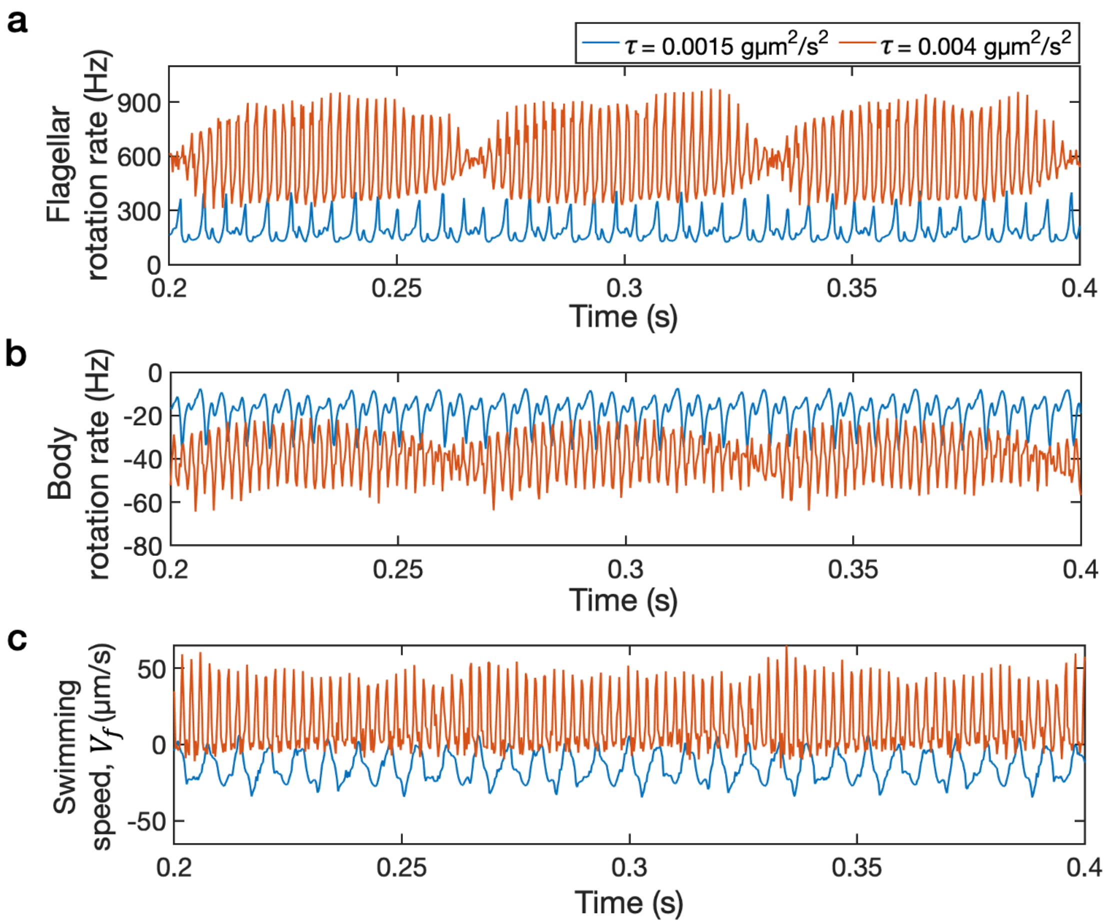

Figure S1: Time evolutions of flagellar rotation rate (top), cell body rotation rate (middle) and swimming speed (bottom) for two overwhirling cases when the applied torque is given as $\tau = 0.0015$ gμm^2/s^2 (blue) and 0.004 gμm^2/s^2 (red). For the small value of $\tau = 0.0015$, the overall swimming direction is backward (negative speed); however, for the large value of $\tau = 0.004$, the cell swims forward (positive speed).

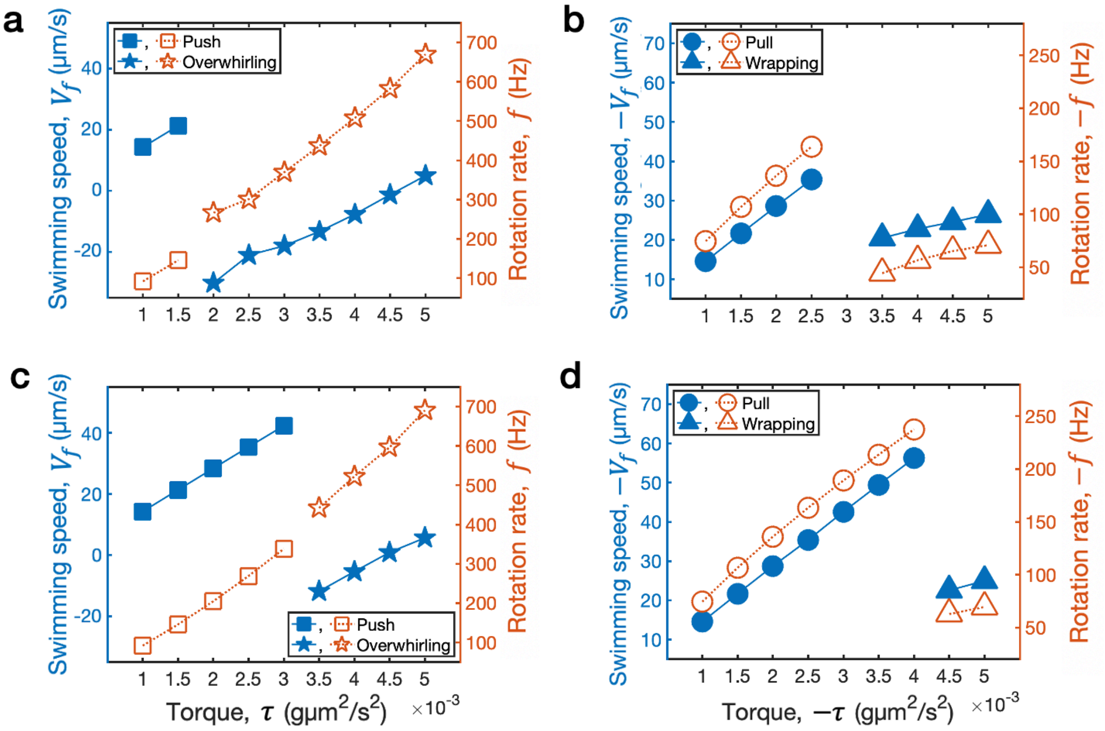

Figure S2: Average swimming speeds and motor rotation rates as functions of the applied torque generated by the flagellar motor. The bending modulus of the filament is fixed as $a = 0.003$ gμm^3/s^2, and the bending modulus of the hook is set as $a^{\text{hook}} = \frac{a}{35}$ (top row) and $a^{\text{hook}} = \frac{a}{15}$ (bottom row). The direction of motor rotation is CCW for left column (a, c) and CW for right column (b, d), where the positive (negative) values of τ lead to CCW (CW) rotation. The motor frequency denoted by f is defined to be positive when the motor turns CCW and vice versa. The forward swimming speed V_f is defined as the velocity of the cell body in the direction from the motor point to the other pole of the cell body. This figure shows, in general, that average swimming speeds and motor rotation rates increase linearly with the increasing applied torque for each mode of motility.

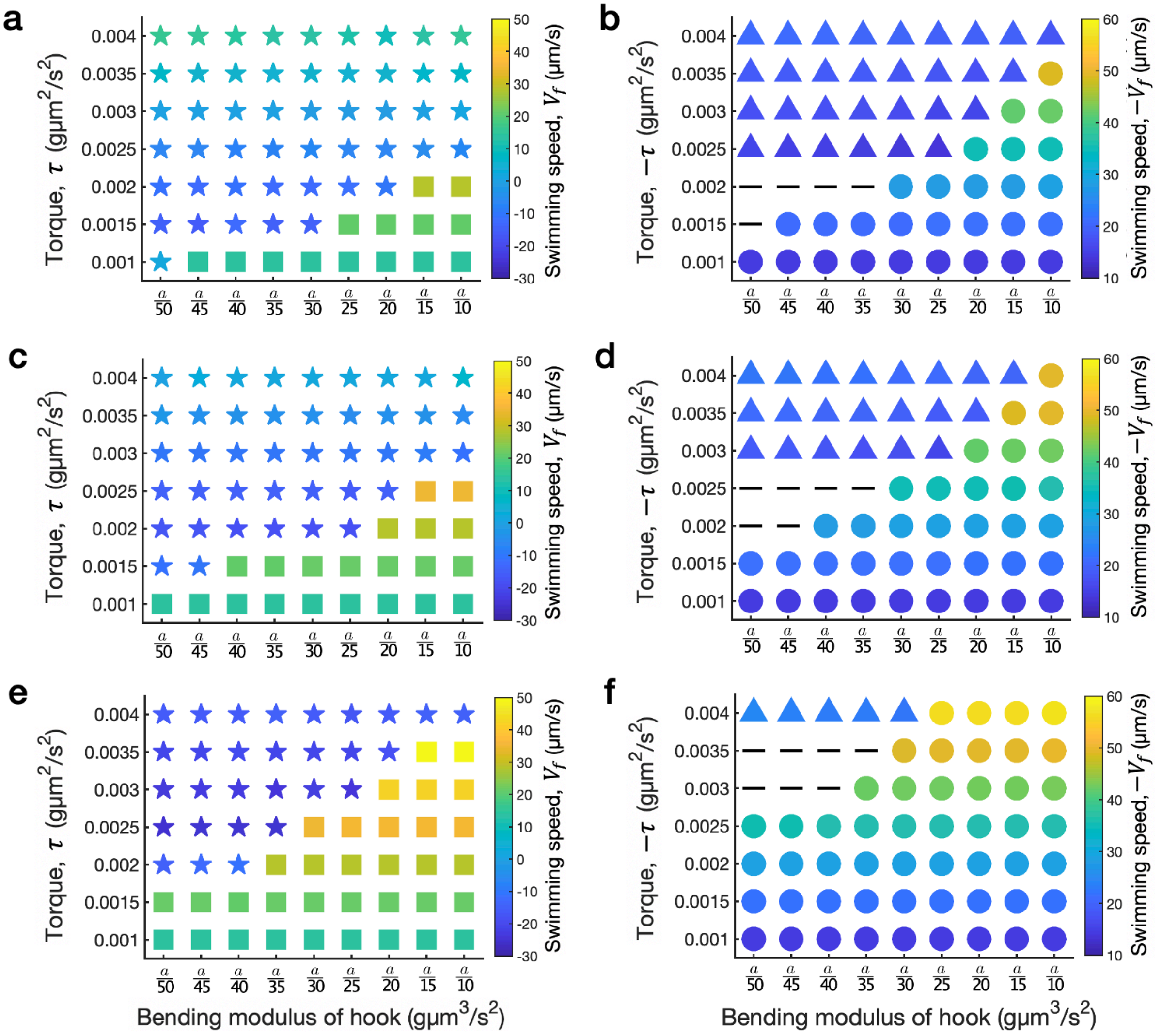

Figure S3: Swimming modes as functions of the bending modulus of the hook (a^{hook}) and the applied torque (τ) when the bending modulus of the filament is fixed at a = 0.002 gμm^3/s^2 (top row), 0.0025 gμm^3/s^2 (middle row) and 0.0035 gμm^3/s^2 (bottom row). In each row, the motor rotates either CCW (left panel) or CW (right panel). Different shapes of markers represent the stable motion of the push (■), overwhirling (★), pull (●), and wrapping (▲) modes. The markers (—) indicate that the bacterium does not show either wrapping or pull mode for a given simulation time, because the hook is too flexible and the applied torque is close to the critical value. Colors indicate the average swimming speeds measured for each mode of motility after the simulation reaches the stable steady motion. For different values of the bending modulus of the flagellar filament, the swimming patterns are similar to each other. However, thresholds that separate push from overwhirling and pull from wrapping are shifted upward as the bending modulus of the filament increases.

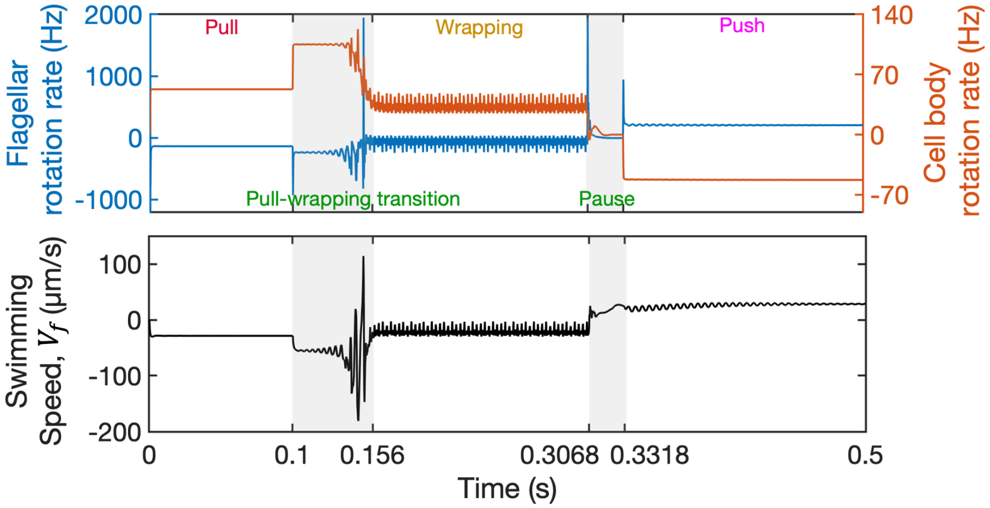

Figure S4: Rotation rates of the motor and the cell body (top) and swimming speed (bottom) of a cell over a sequence of pull, wrapping, pause, and push modes. Each mode is derived by implementing the applied torque as in Fig. 4(a) of the main text. Positive and negative values of flagellar rotation rates correspond to CCW and CW rotation, respectively. Moreover, the cell body counterrotates to the flagellum. If the swimming speed (V_f) is positive, the cell swims forward, and if it is negative the cell swims backward.

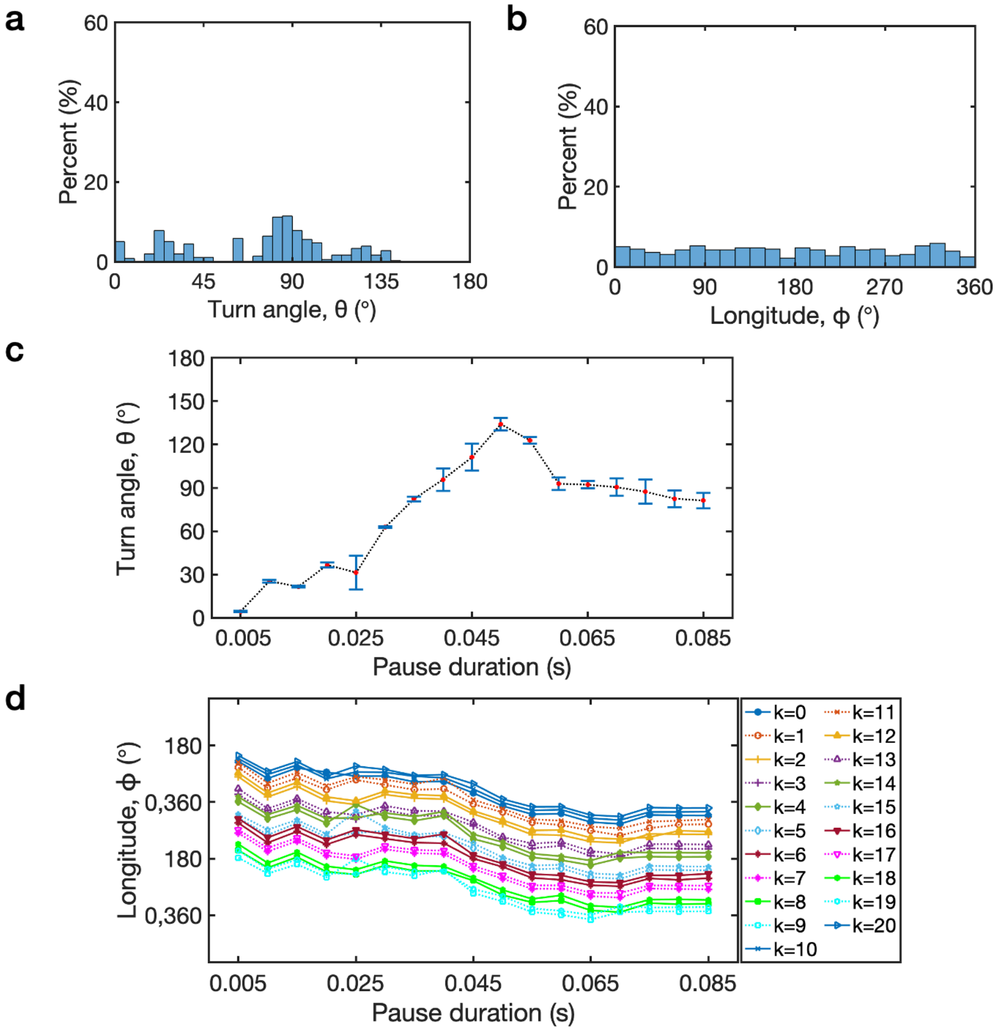

Figure S5: Effect of a pause between wrapping modes on cells' reorientation: wrapping-pause-wrapping. For each fixed pause duration, pause initiation time varies from $t = 0.15$ s up to $t = 0.184$ s with the uniform time interval of 0.0017 s, i.e., $t = 0.15 + 0.0017k$ for $k = 0, 1, ..., 20$, totaling 21 cases. The pause duration also varies from 0.005 s to 0.085 s with the time interval of 0.005 s. Histograms display distributions of (a) turn angles and (b) longitudes of cells resulting from 357 combinations of pause durations and initiation times. The distribution of turn angles shows two noticeable peaks around $25° - 30°$ and $85° - 90°$, whereas the longitudes show an approximately uniform distribution. In (c), the mean (red dot) and the standard deviation of turn angles are measured for each pause duration. The turn angle ranges from $4°$ to $140°$ as pause duration changes, but it remains approximately constant to $85° - 90°$ when the pause duration is longer than 0.06 s. In (d), for each pause duration, the longitude ranges from $0°$ to $360°$ as the initiation time varies over a cycle of flagellar rotation.

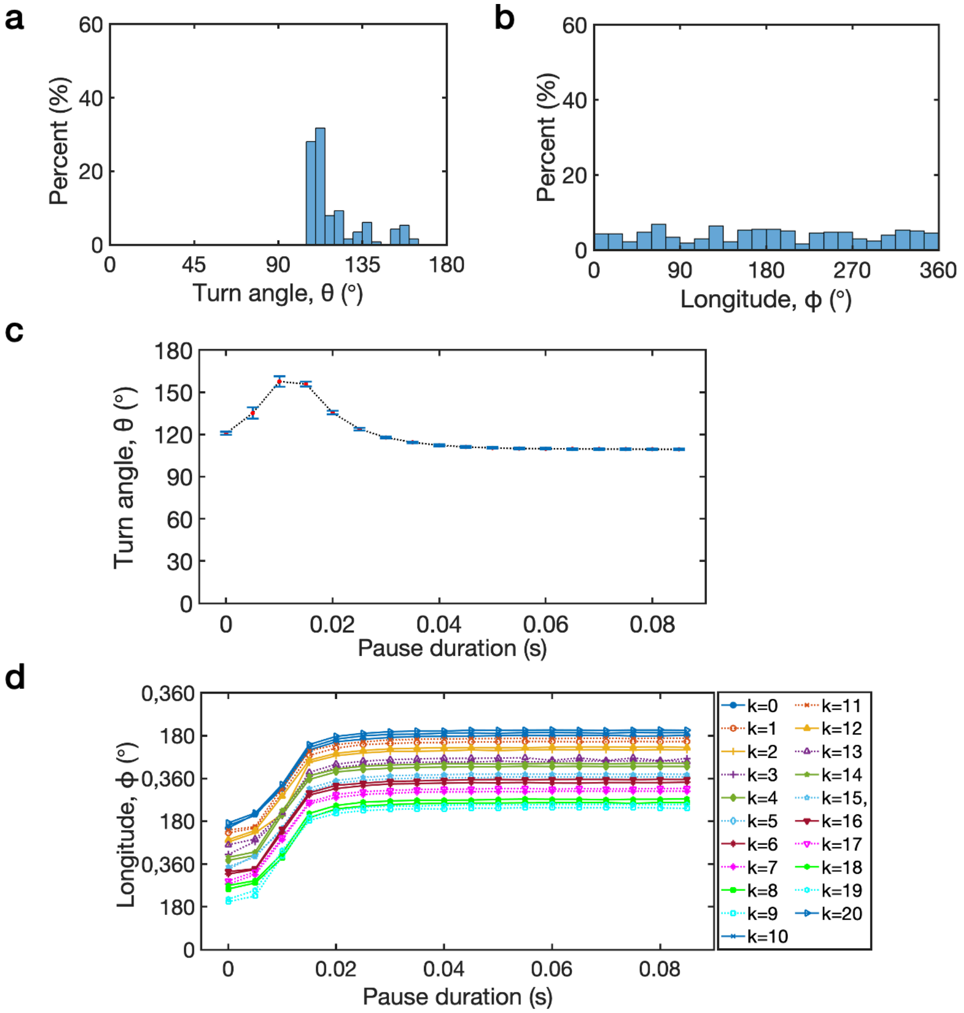

Figure S6: Turn angles and longitudes of cells swimming via wrapping-pause-pull modes. Histograms display distributions of (a) turn angles and (b) longitudes of cells, when 378 combinations of different pause start times and durations are considered: pause starts at $t = 0.15 + 0.0017k$ for $k = 0, 1, ..., 20$ and pause duration varies from 0 s to 0.085 s. Note that the pause duration of 0 s represents events for no pause. The turn angles show a unimodal distribution peaked around 110°, but the longitude has spread out from 0° to 360°. For each pause duration, the mean and the standard deviation of turn angles are shown in (c), which demonstrates that turn angles do not change much if the pause duration is long enough. Similarly, the longitudes remain approximately constant with sufficiently long pause duration, see (d). For each fixed pause duration, the longitude ranges from 0° to 360° as the pause initiates at different times during a cycle of flagellar rotation.

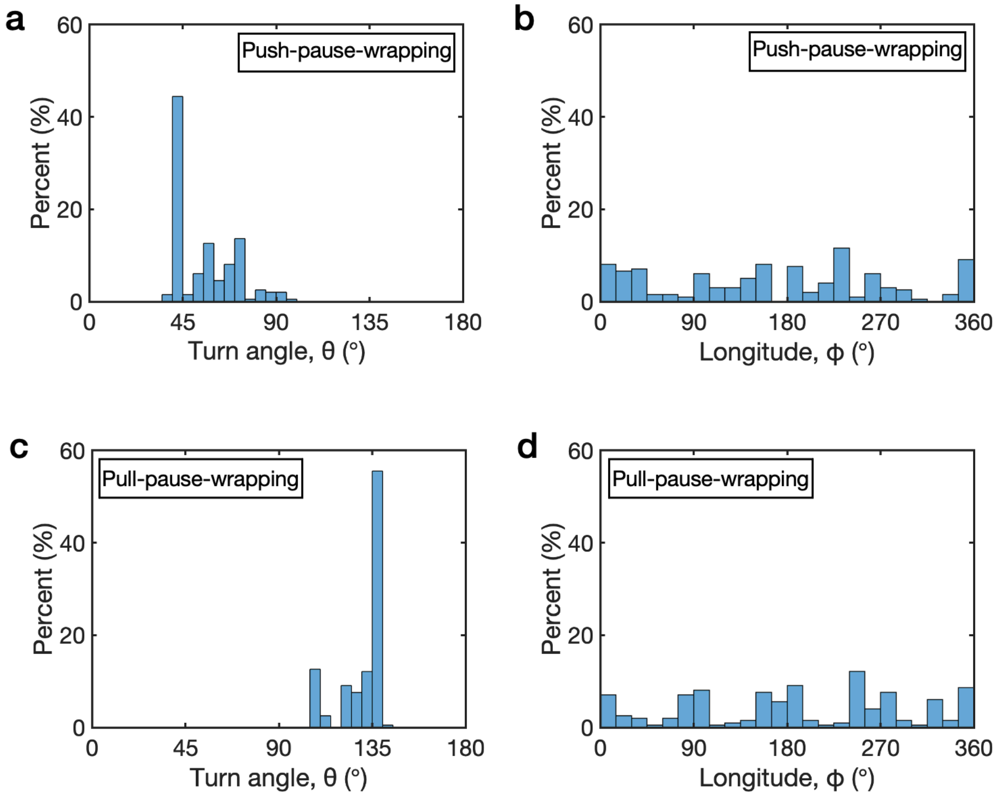

Figure S7: Histograms of turn angles (left panels) and longitudes (right panels) of cells experiencing push-pause-wrapping modes (top panels) and pull-pause-wrapping modes (bottom panels). Each histogram is built from 198 combinations of pause initiation times and pause durations as follows: the pause duration varies from 0 s to 0.085 s and the initiation time for pause varies from 0.15 s to 0.169 s with uniform time interval of 0.0019 s. The turn angles (a, c) show unimodal distributions, centered at $40° - 45°$ (a) and $135° - 140°$ (c), and the averages of those turn angles are $55.0° \pm 14.9°$ and $131.0° \pm 10.3°$, respectively. The longitudes (b, d) take multimodal distributions.

**Description (parameter)**	**Value**
Mesh width for flagellum (Δs)	0.0303 μm
Helical radius of filament (R)	0.35 μm
Helical pitch of filament (P)	2 μm
Axial length of helical filament (L_f)	3.5 μm
Curvilinear length of filament (ℓ)	4.99 μm
Intrinsic curvature of filament ($\Omega_1 = \Omega_2$)	1.5637 μm^{-1}
Intrinsic twist of filament (Ω_3)	1.4222 μm^{-1}
Bending modulus of filament ($a_1 = a_2 = a$)	0.003 gμm^{3}s^{-2}
Twist modulus of filament (a_3)	0.003 gμm^{3}s^{-2}
Shear modulus ($b_1 = b_2$)	2 gμms^{-2}
Stretch modulus (b_3)	2 gμms^{-2}
Length of hook (L_h)	3Δs
Bending modulus of hook ($a_{1,2}^{\mathrm{hook}} = a^{\mathrm{hook}} = a/25$)	0.00012 gμm^{3}s^{-2}
Twist modulus of hook ($a_3^{\mathrm{hook}} = a_3$)	0.003 gμm^{3}s^{-2}
Torque (τ)	0.002 (push), -0.002 (pull), -0.004(wrapping) gμm^{2}s^{-2}
Cell body length ($2L_b$)	2.0 μm
Cell body width ($2R_b$)	0.9 μm
Penalty parameter (K)	2 gs^{-2}
Fluid viscosity (μ)	0.01×10^{-4} g(μm s)$^{-1}$
Parameter for repulsive force (C)	2×10^3 gs^{-2}
Minimum distance allowed between the filament and the cell body (D)	0.1 μm
Regularization parameter (ε)	3Δs
Translational drag coefficient (α_1)	1.212×10^{-7} g(μm s)$^{-1}$
Translational drag coefficient (α_2)	4×10^{-6} gs^{-1}
Rotational drag coefficient (β)	1.212×10^{-7} gμm s^{-1}
Time step (Δt)	2×10^{-8} s

Table S1: Computational and physical parameters

L_{h} / $\tau(\mathbf{g\mu m^2/s^2})$	0	Δs	$2\Delta s$	$3\Delta s$ (default)	$4\Delta s$	$5\Delta s$	$6\Delta s$
0.004	push	overwhirling	overwhirling	overwhirling	overwhirling	overwhirling	overwhirling
0.003	push	push	overwhirling	overwhirling	overwhirling	overwhirling	overwhirling
0.002	push	push	push	push	overwhirling	overwhirling	overwhirling
-0.004	pull	pull	pull	wrapping	wrapping	wrapping	wrapping
-0.005	pull	pull	wrapping	wrapping	wrapping	wrapping	wrapping
-0.006	pull	wrapping	wrapping	wrapping	wrapping	wrapping	wrapping

Table S2: Effect of the hook length on swimming modes

Video S1: Swimming modes of a polarly-flagellated bacterium whose helical filament is intrinsically left-handed. Each row shows two stable motions when the motor rotates either CCW (top panel) or CW (bottom panel). These two stable motions are separated by the thresholds of applied torque, τ_{ccw} or τ_{cw}, respectively. When the applied torque $|\tau|$ is below $|\tau_{\mathrm{ccw}}|$ or $|\tau_{\mathrm{cw}}|$, a cell exhibits push or pull mode, respectively (left panel). When $|\tau|$ is above $|\tau_{\mathrm{ccw}}|$ or $|\tau_{\mathrm{cw}}|$, a cell undergoes overwhirling or wrapping mode, respectively (right panel).

Video S2: A series of swimming modes in the order of pull, wrapping, pause, and push modes. The colored path is a trajectory of the centroid of the cell body (left panel) as the cell consecutively experiences swimming modes in accordance with the applied torque of the motor in time (right top panel). The corresponding swimming speed over time is shown in the right bottom panel.

Video S3: Effect of a pause on cells' reorientation when cells go through wrapping-pause-push modes. The left panel displays the cases with four different pause initiation times, $\mathscr{P}_{\mathrm{I}}^{k} = (0.15 + 0.0017k)$ s for $k = 0, 3, 6, 9$, while the pause duration is fixed as $\mathscr{P}_{\mathrm{D}}^{7} = 0.06$ s. The right panel shows the cases with four different pause durations, $\mathscr{P}_{\mathrm{D}}^{j} = (0.025 + 0.005j)$ s for $j = 0, 1, 4, 7$, while the pause initiation time is fixed as $\mathscr{P}_{\mathrm{I}}^{0} = 0.15$ s. Four different simulations in each panel are shown simultaneously, while all cells take the same path until 0.15 s and they change their trajectories depending on the pause duration or pause initiation time.

Video S4: Swimming trajectories of cells going through a wrapping mode near the wall. We consider four cells with respect to four combinations of two different initial cell rotation angles (180°, 337.5°) and two different initial heights ($h_0 = 1.0,\ 2.5\ \mu$m), and place them parallel to the wall. Four different simulations are simultaneously displayed when $\tau = -0.004$ gμm^2/s^2 is set for all times.

Video S5: Escaping cells from the wall by switching swimming mode to a wrapping mode. When a cell settles down and is trapped near the wall during a pull mode (left panel) or a push mode (right panel), switching to a wrapping mode helps the cell to escape from the wall.
