# Supplementary figures and images for: Modeling of lophotrichous bacteria reveals key factors for swimming reorientation
Source: Sci Rep. 2022 Apr 20;12:6482. doi: 10.1038/s41598-022-09823-4 (PMC9021275; doi:10.1038/s41598-022-09823-4)

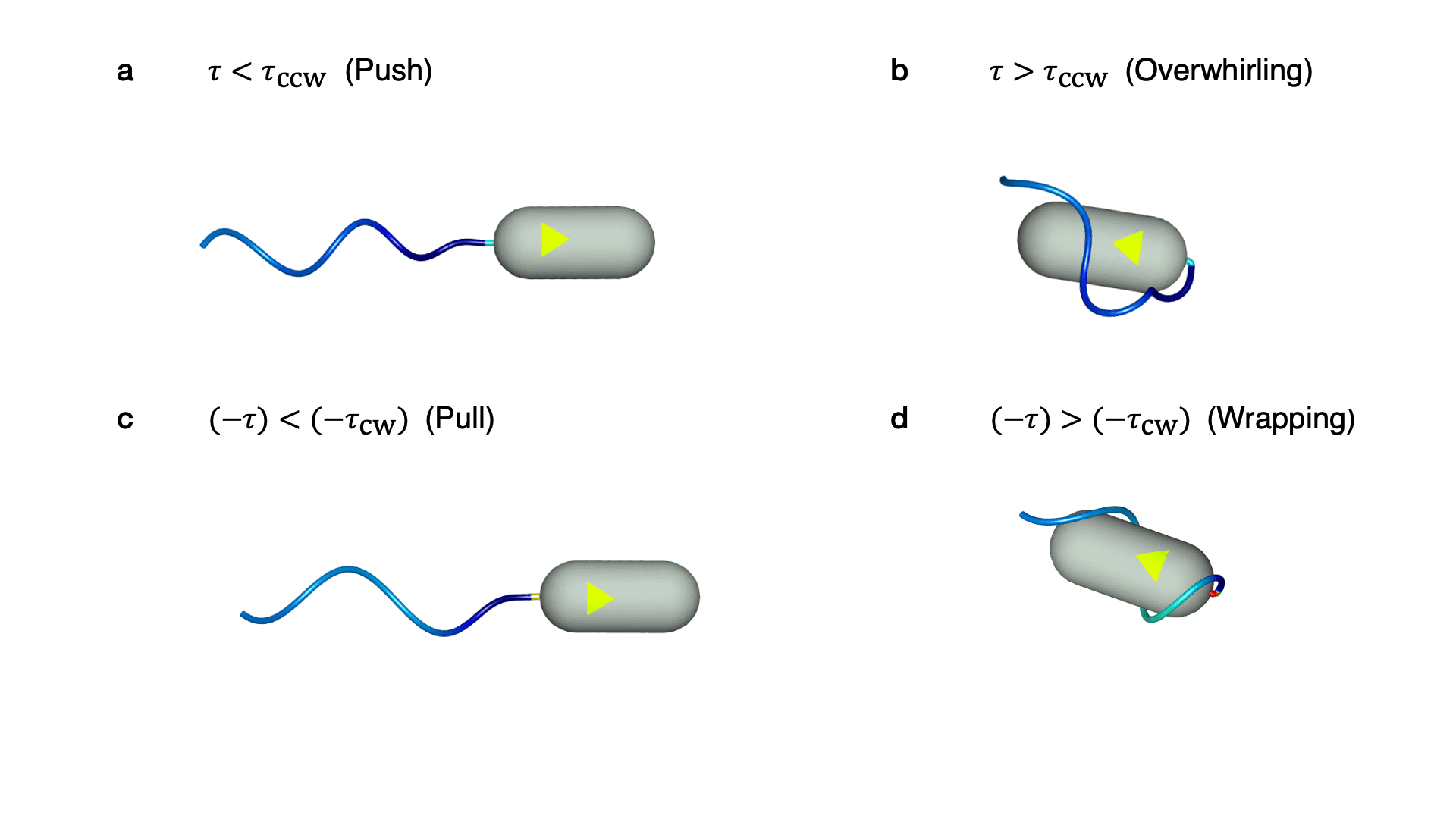

Supplement: Supplementary file 2 — Supplementary Information 2. [file 41598_2022_9823_MOESM2_ESM.gif]

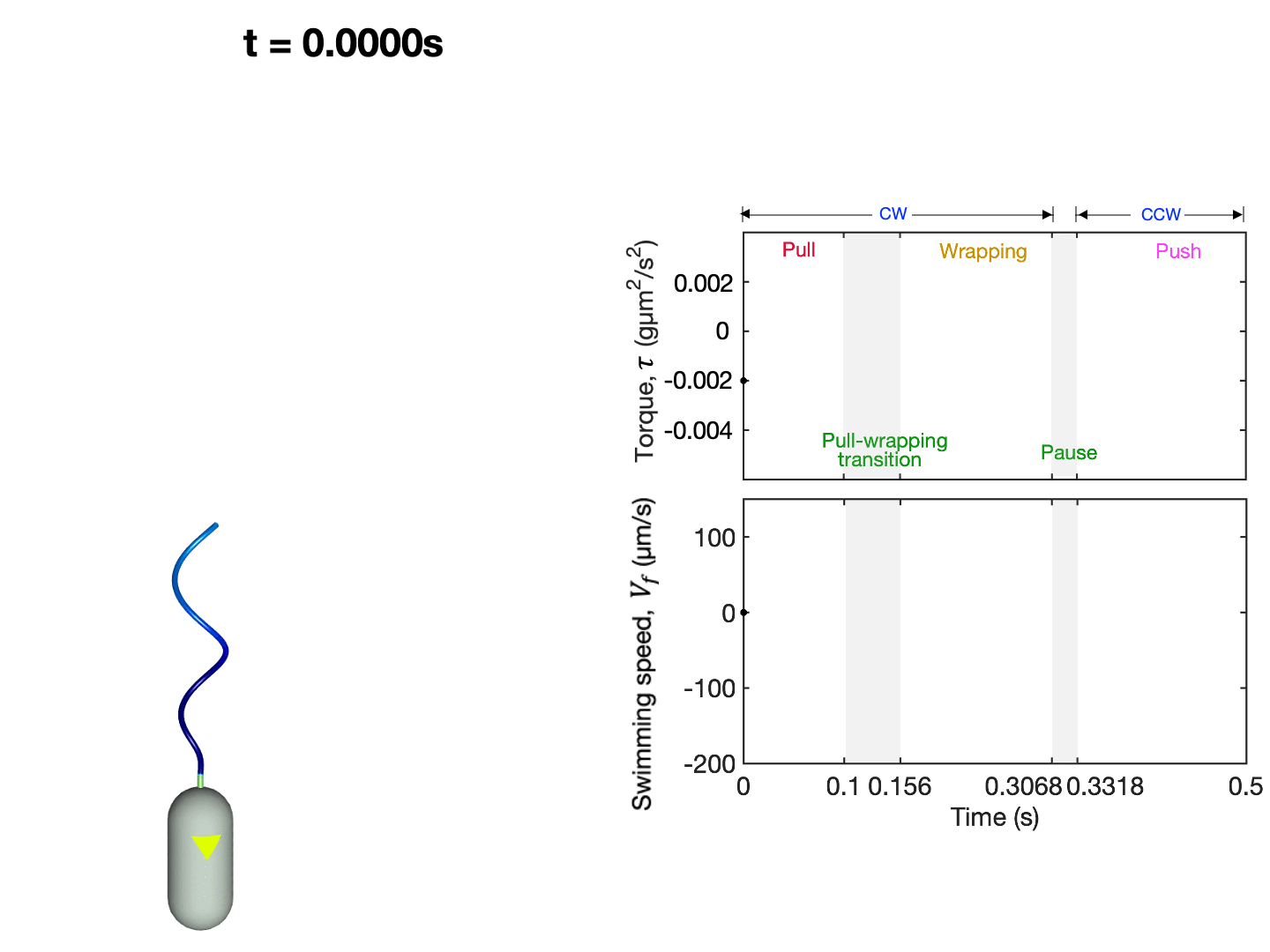

Supplement: Supplementary file 3 — Supplementary Information 3. [file 41598_2022_9823_MOESM3_ESM.gif]

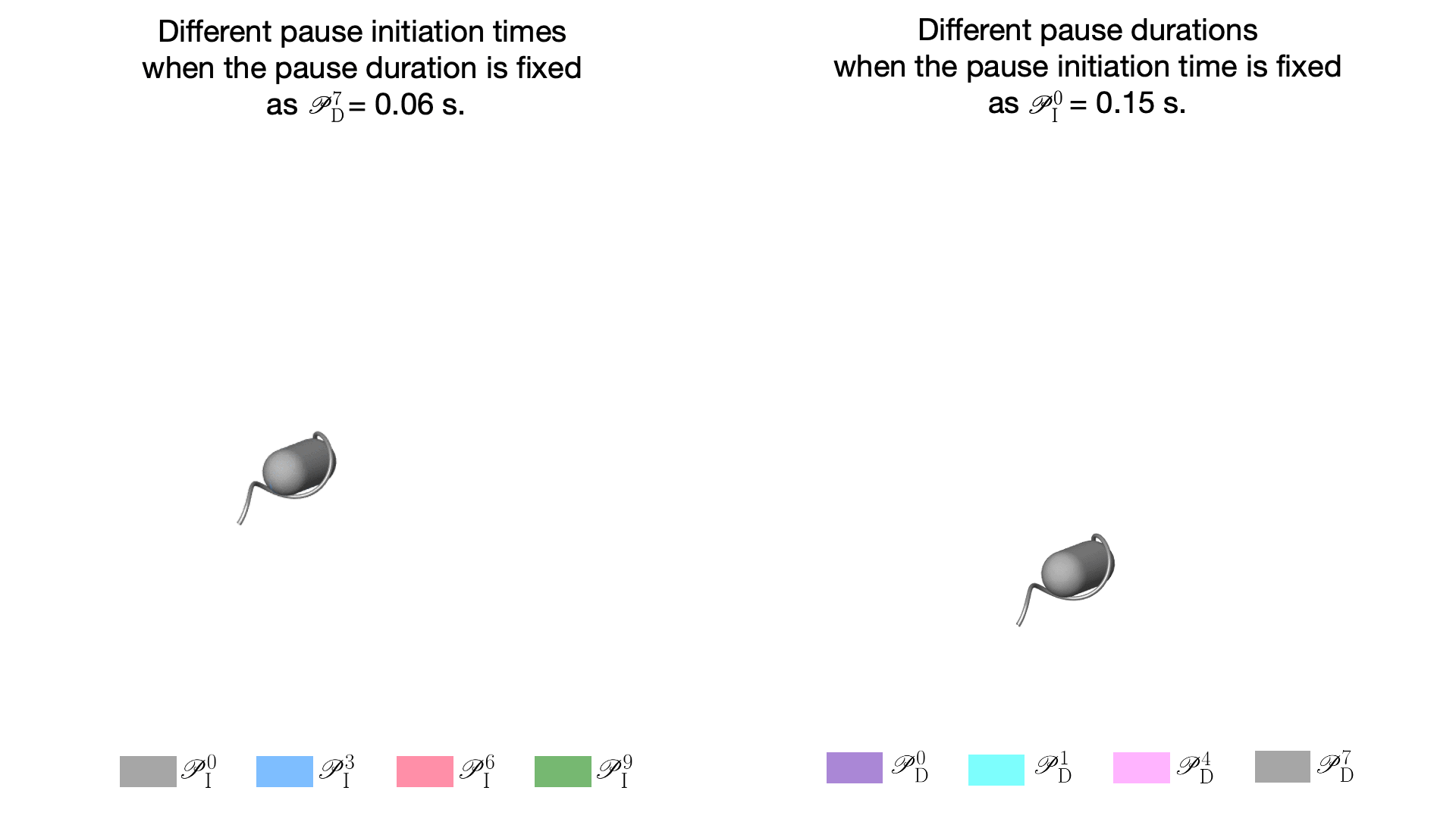

Supplement: Supplementary file 4 — Supplementary Information 4. [file 41598_2022_9823_MOESM4_ESM.gif]

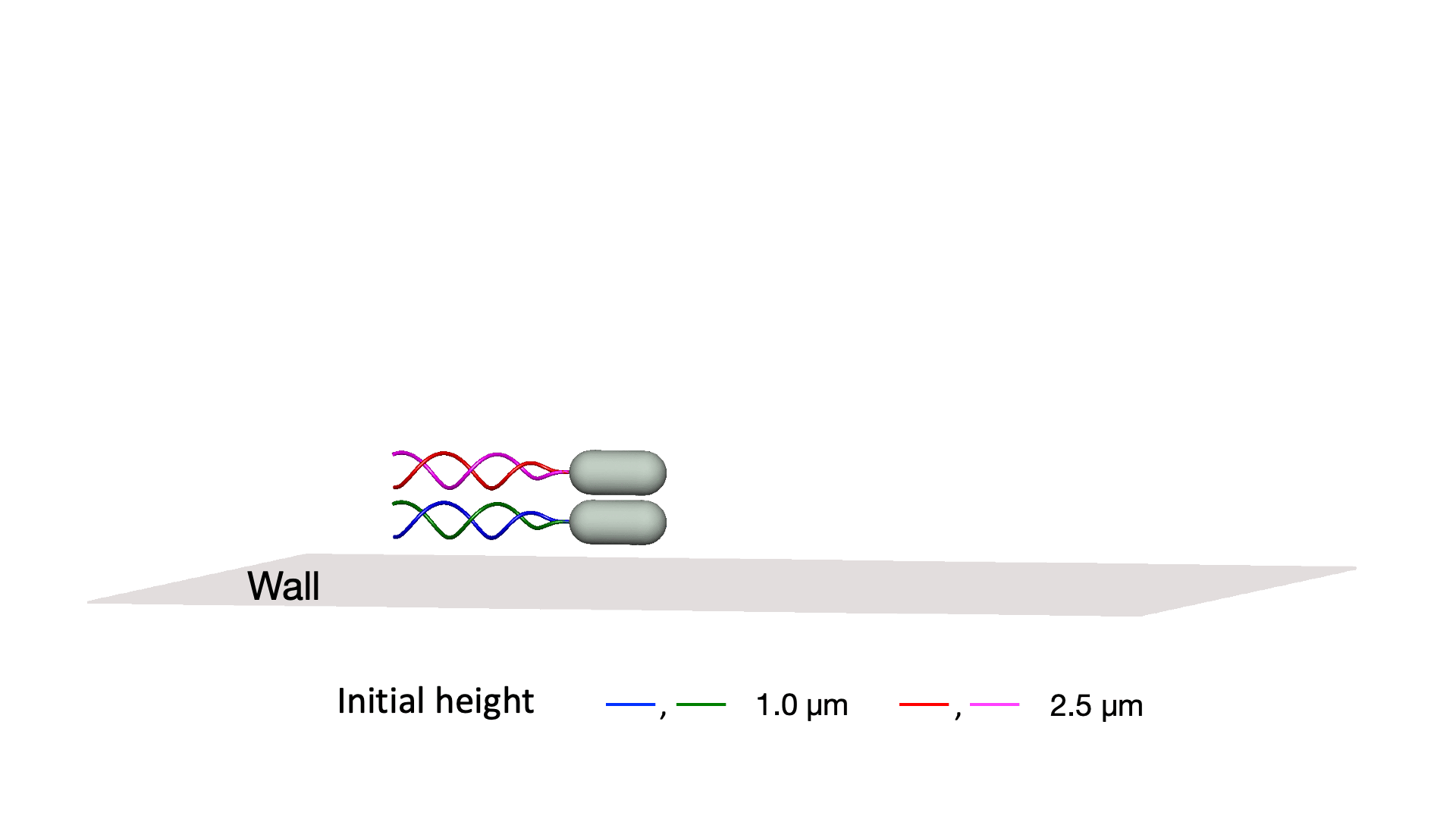

Supplement: Supplementary file 5 — Supplementary Information 5. [file 41598_2022_9823_MOESM5_ESM.gif]

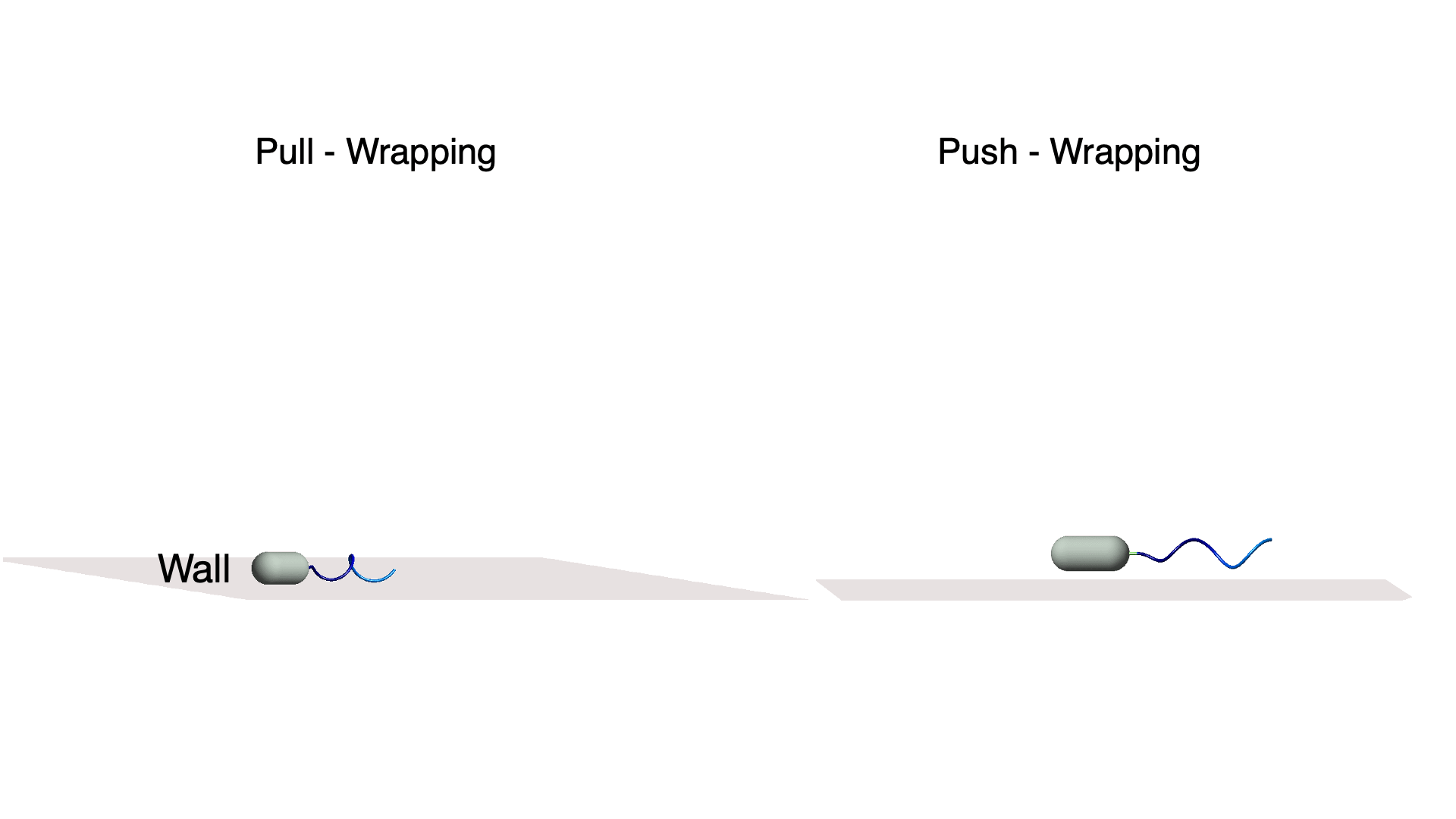

Supplement: Supplementary file 6 — Supplementary Information 6. [file 41598_2022_9823_MOESM6_ESM.gif]
